# Nanographenes and Graphene Nanoribbons as Multitalents of Present and Future Materials Science

**DOI:** 10.1021/jacs.2c02491

**Published:** 2022-06-07

**Authors:** Yanwei Gu, Zijie Qiu, Klaus Müllen

**Affiliations:** † Max Planck Institute for Polymer Research, Ackermannweg 10, 55128 Mainz, Germany; ‡ Institute for Physical Chemistry , Johannes Gutenberg University Mainz, Duesbergweg 10-14, 55128 Mainz, Germany; § Shenzhen Institute of Aggregate Science and Technology, School of Science and Engineering, The Chinese University of Hong Kong, Shenzhen 518172, China

## Abstract

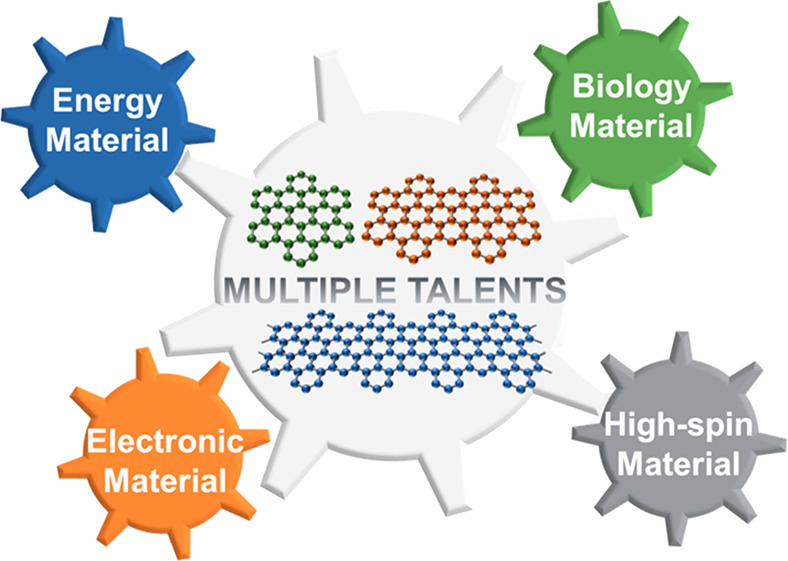

As cut-outs from a graphene sheet, nanographenes (NGs) and graphene nanoribbons (GNRs) are ideal cases with which to connect the world of molecules with that of bulk carbon materials. While various top-down approaches have been developed to produce such nanostructures in high yields, in the present perspective, precision structural control is emphasized for the length, width, and edge structures of NGs and GNRs achieved by modern solution and on-surface syntheses. Their structural possibilities have been further extended from “flatland” to the three-dimensional world, where chirality and handedness are the jewels in the crown. In addition to properties exhibited at the molecular level, self-assembly and thin-film structures cannot be neglected, which emphasizes the importance of processing techniques. With the rich toolkit of chemistry in hand, NGs and GNRs can be endowed with versatile properties and functions ranging from stimulated emission to spintronics and from bioimaging to energy storage, thus demonstrating their multitalents in present and future materials science.

## 1 Introduction

Among classics of chemistry, the structure and properties of benzene come to mind, and aromaticity, despite or perhaps because of its somewhat diffuse definition, still ignites lively discussions.^1−3^ Those who fancy elegant multistep syntheses with new stereogenic centers may tend to look down at “flat” benzene structures. Many pharmaceuticals, however, are made with benzene functionalization as a key step.^4^ More importantly, the conjugated hexagon in benzene is a versatile module for the design of complex molecules such as linear oligophenylenes or disc-type polycyclic aromatic hydrocarbons (PAHs). These are by no means lacking the appeal of chirality, as is obvious from cases of atropisomerism and helicity,^5−7^ and there are many good reasons to even let them grow into helical polymers.

Rational assembly of regular building blocks by covalent or noncovalent bonding has become a widely employed protocol of modern chemistry, and this modular concept has made benzene an indispensable element in nanoscience and materials science. Further, the ability to visualize and manipulate nanosized molecules by scanning probe methods has stimulated a systematic increase in the size of “benzene” nanostructures in one, two, or three dimensions (1D, 2D, or 3D). A good case is that of hexa-*peri*-hexabenzocoronenes (HBCs) as soluble “superbenzenes,” for which early studies with scanning tunneling spectroscopy have allowed recording of current–potential curves at the single-molecule level on the path to emerging nanoelectronics.^8,9^

**Scheme 1:**
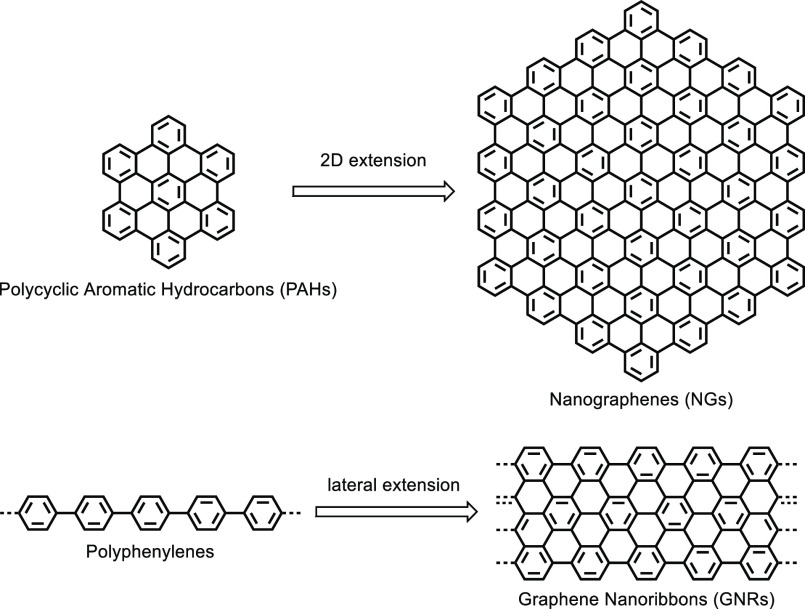
Representative Examples of Benzene-Based Graphenic Molecules

In the past decades, the world of carbon-rich polyphenylenes and PAHs has been extended toward carbon allotropes such as fullerenes, carbon nanotubes (CNTs), and graphene. While representing different dimensionalities, they are all made up of fused benzene rings and, remarkably enough, connect the realm of molecules with those of discrete particles and bulk materials. Our title compounds are nanographenes (NGs), ultralarge PAHs, and graphene nanoribbons (GNRs), ladder-type polyphenylenes (Scheme 1). Quite different ways of approaching GNRs are possible since they can be regarded either as (1) polyphenylenes extended laterally, (2) large PAHs grown into 1D, or (3) cut-outs from a graphene lattice.^10^ Indeed, various subunits have been carved out of graphene flakes by electron-beam lithography, while GNRs have been produced by slicing or squashing CNTs.^11−13^ The structural versatility of GNRs is outstanding considering not only variations of their length, width, and edge structure,^14−18^ but also heteroatom incorporation,^19−21^ nonplanarity, and helicity,^22−26^ as well as “drilling” of holes.^27−29^

These features are demanding challenges for synthesis, especially when structural precision is a major necessity. Carbon nanostructures such as NGs and GNRs hold promise for unprecedented physical properties, from exotic quantum states^30,31^ to stable biexcitonic states,^32^ and from spin transport^33^ to magnetism.^34,35^ Toward that end, GNRs must be made both structurally perfect and also narrow enough, and these two characteristics highlight the role of organic synthesis in graphene materials science. Solid-state and thin-film structures become important as well, and this emphasizes the importance of processing techniques such as gas-phase deposition or shear-mix exfoliation. Good cases include single sheets of NGs and GNRs, which are needed for defined van der Waals heterostructures with 2D materials.^36^

The vitality of research into NGs and GNRs is readily proven by the significant attention directed from various fields of chemistry, physics, biology, and materials science. What this perspectives article is meant to demonstrate is that precisely synthesized NGs and GNRs are astonishing multitalents in the field of functional carbon nanostructures.

## 2 Very Large – However, Still Perfect?

GNRs are defined as ribbon-shaped, quasi-1D graphenic nanostructures with aspect ratios larger than 10.^37^ From the viewpoint of polymer science, GNRs can be regarded as multistranded ladder polymers whose thermal and mechanical properties are expected to differ substantially from those of traditional single-stranded polymers.^10,38,39^ Additionally, various conjugation pathways arising in GNRs hold promise for special electronic band structures.^40,41^ Indeed, in the wake of the graphene hype, GNRs have attracted considerable attention from solid-state physics and materials science, which had an important electronic basis: despite its high charge carrier mobility, the vanishing band gap of graphene excluded widespread application as the semiconductor of field-effect transistors (FETs) due to unavoidable off-currents.^42,43^ In contrast, the geometric confinement prevailing in GNRs holds promise for finite and controllable band gaps. Theoretical studies of GNRs have revealed that their electronic properties, including band gaps and charge-carrier mobilities, depend critically on their width and edge structures.^15,40,44,45^ Materials scientists, recognizing the immense appeal of GNRs, have then employed various harsh methods of synthesis, including (1) lithographic^46^ and metal-nanoparticle catalyzed^47^ cutting of graphene sheets; (2) sonochemical extraction from expanded graphite;^37^ and (3) unzipping,^48^ plasma etching,^11^ and high-pressure squashing^13^ of CNTs. These methods have found appreciable attention, but lack the structural perfection needed for reliable band gap engineering. Precision polymer synthesis is therefore brought into play.

**Scheme 2:**
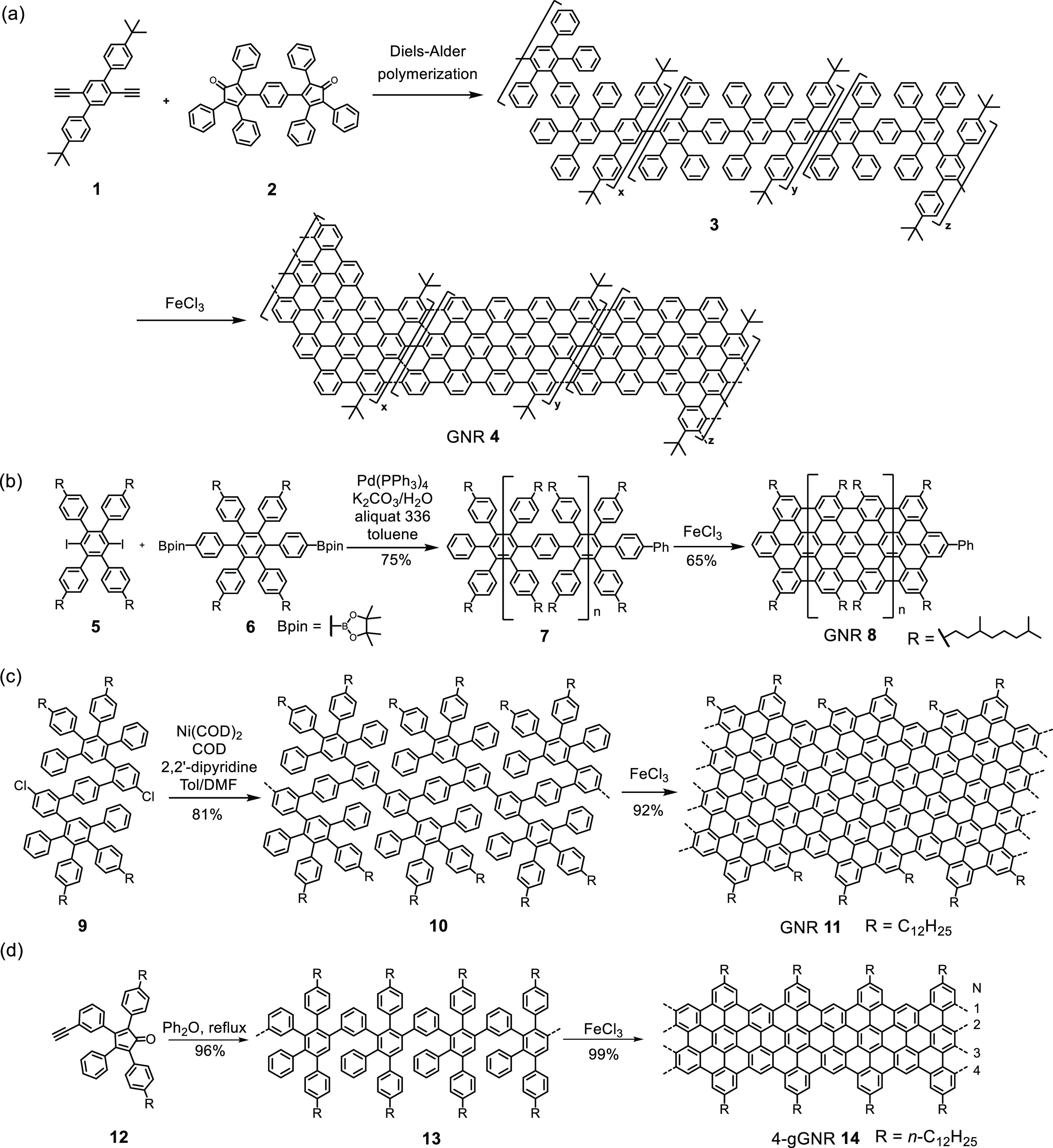
(a) Solution-Mediated Synthesis of GNR **4** through A_2_B_2_-Type Diels–Alder Polymerization;^54^ (b–c) Transition-Metal-Catalyzed Polycondensations;^52,53^ and (d) Diels–Alder Cycloadditions Used To Realize GNRs **8**, **11**, and **14** in Solution^55^

Synthesis of GNRs by consecutive fusion of small PAHs is unrealistic. Of widespread current use is a “polymerization–graphitization” protocol,^49^ in which branched polyphenylenes are made in a first step and then subjected to a chemical cyclodehydrogenation. Now the scope of cyclodehydrogenation is further broadened by the recent success of electrochemical methods.^50,51^ The branched polyphenylenes serve as carbon reservoirs; therefore, their topologies are crucial since the flattening process should neither leave holes of partially dehydrogenated spots nor produce spatial overlap of benzene rings. Scheme 2 presents some precursor polymers which document both the importance of a multibenzene “Lego” and the need for high molecular weights in the precursors.^52−55^ Transition-metal-catalyzed polycondensations, as demonstrated in Scheme 2b–c, suffer from unavoidable loss of functional groups,^52−54^ thus disturbing the perfect stoichiometries required for polycondensation and limiting the molecular weights, while repetitive Diels–Alder cycloadditions according to Scheme 2d can provide the targeted lengths of the polymers. The trick is to use an AB-type monomer **12** which contains the conjugated diene and an ethynyl group functioning as a dienophile. The structure of the resulting gulf-edged GNR **14** (4-gGNR, where “4” is the ribbon width defined by the number of carbon atoms across the ribbon) was firmly verified by infrared, Raman, ultraviolet–visible absorption, and nuclear magnetic resonance (NMR) spectroscopies, and an astonishing length of 600 nm was determined from dynamic light scattering experiments.^55^ Generating ultralarge GNRs via subsequent cyclodehydrogenation proceeds with a high degree of conversion and, surprisingly enough, provides solution-processable materials. This GNR synthesis certainly pushes the limits of molecular-based material synthesis and has been taken up by many research groups.^56−59^

**Scheme 3:**
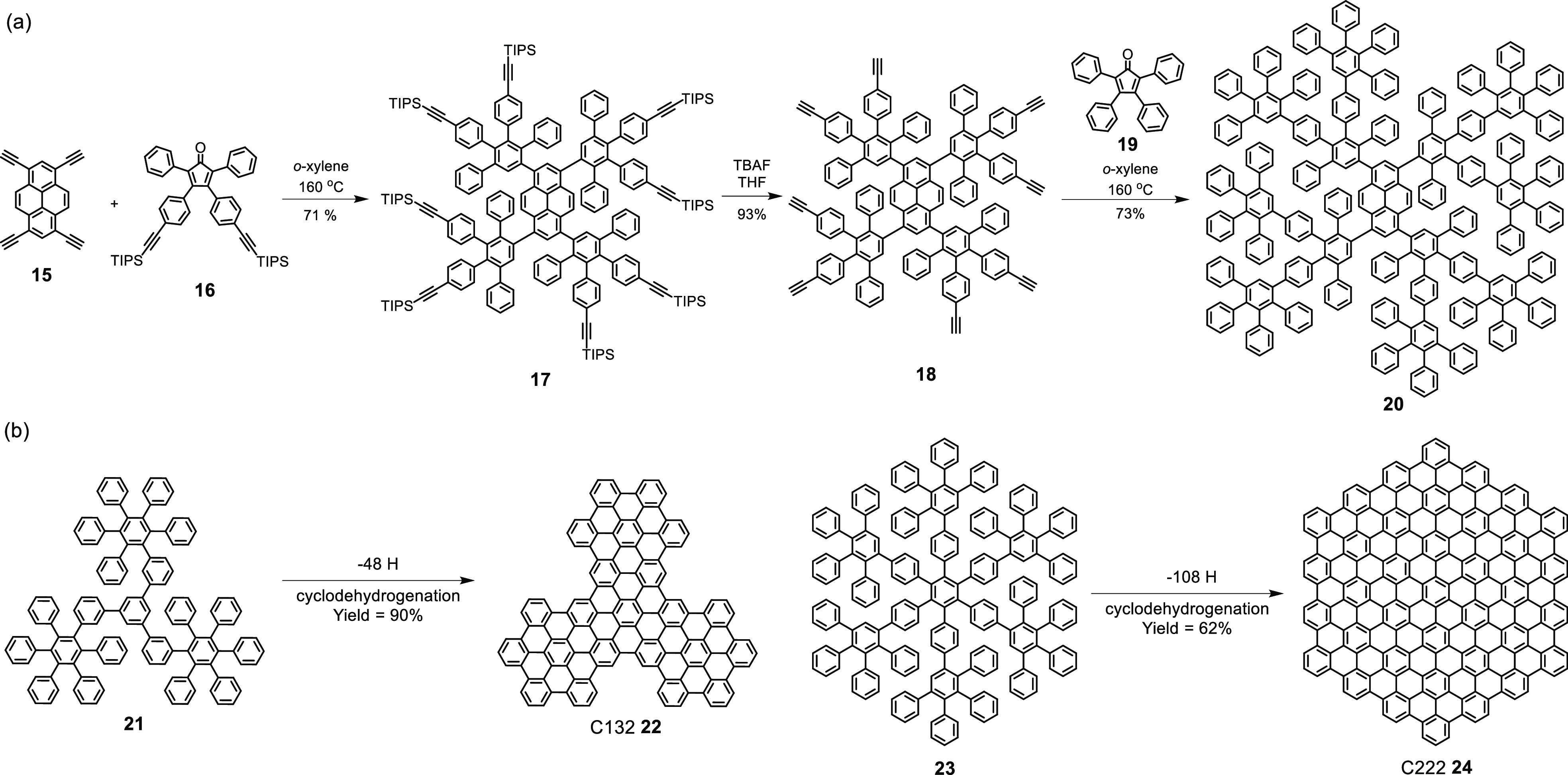
(a) Synthesis of PPD **20** from AB_2_-Type Branching Reagent **16**;^60^ (b) Syntheses of C132 and C222 by Cyclodehydrogenation from Relatively Small Dendrimers **22** and **24** with High Yields^64,65^

The precursor polyphenylenes have multiple branches, but some of them are even dendritic. It is amazing that in seeking to extend PAHs into NGs, a new generation of dendrimers has been developed as polyphenylene dendrimers (PPDs) which consist only of twisted benzene units. Similar to the polyphenylene precursor **13**, PPDs are also synthesized by repetitive Diels–Alder cycloadditions, but require a diethynyl-functionalized tetraphenylcyclopentadienone as an AB_2_-type branching reagent that carries two dienophilic units (Scheme 3a).^60^ Of course, the stepwise growth of higher dendrimer generation requires a protection–deprotection sequence, but it is the perfection of this protocol that guarantees high purity of PPDs as monodisperse polymers. The initial members of the series, as starting points for cyclodehydrogenation, are relatively small, as shown for C132 **22** and C222 **24** (Scheme 3b), but homologous dendrimers can be built up to the ninth generation with molecular weights of 1.9 MDa.^61,62^ There are many possible extensions into other fields of materials chemistry. One example is the transformation of PPDs with peripheral oligothienyl arms into networks upon electrochemical oxidative coupling.^63^ At more positive potentials, the dendrimer cores undergo partial flattening toward graphenic structures, which causes a dramatic increase in the electrical conductivity of the “graphene–thiophene” hybrid.

Returning to the role of branched polyphenylenes as carbon reservoirs for precise synthesis of graphenic molecules, the critical step is cyclodehydrogenation to provide the much-needed quantitative flattening. Model reactions for small oligomers have led the way for investigations of cyclodehydrogenation efficiency. Astonishingly, far beyond the transformation of hexaphenylbenzene toward HBC, larger and larger precursors proceed with extremely high yields (Scheme 3b).^64−66^ Purification of such large NGs, which are practically insoluble, can only be done by washing with organic solvents to remove soluble precursors and byproducts, so it is critical to monitor the cyclodehydrogenations by high-resolution mass spectrometry. HBC, the starting member, can now be found as a widely accepted building block for an increasing number of complex unsaturated hydrocarbons used as chromophores and organic semiconductors.^67−75^

**Scheme 4:**
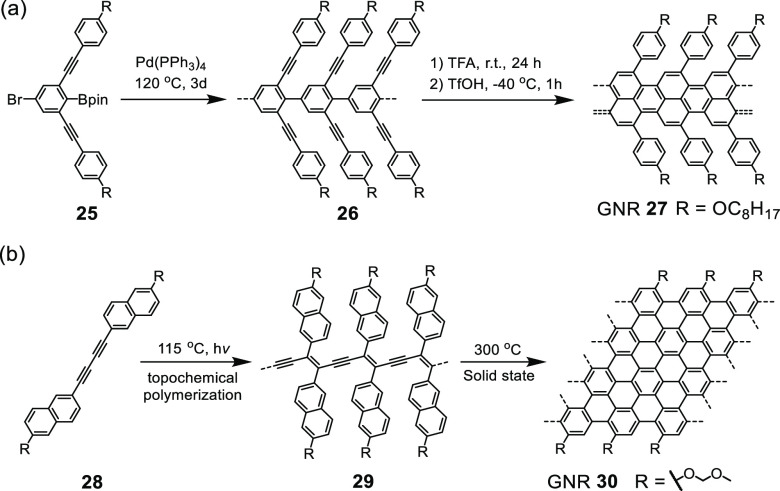
GNRs **27** and **30** Synthesized by (a) Brønsted Acid Promoted Nonoxidative Benzannulation^89^ and (b) Topochemical Polymerization^90^

The Scholl reaction has played an indispensable role in quantitative cyclodehydrogenations of suitable precursors toward NGs and GNRs in solution, although sometimes unexpected rearrangements and/or inclusion of undesired halogens may occur.^76−80^ Alternatively, photocyclization of stilbene and related compounds,^81^ as well as annulative π-extension and dehydrative π-extension reactions,^82−84^ have been introduced as valuable tools for precise syntheses of larger and larger PAHs. With installation of halogens in the precursors at the ring-closing positions, cyclization can be further facilitated through intramolecular aryl–aryl coupling,^85,86^ but such halogenated precursors are synthetically more demanding. Efficient regioselective zipping of carbon–fluorine bonds via cyclodehydrofluorination on alumina provides new mechanisms for cyclizations providing PAHs and NGs.^87,88^ In addition to the “polymerization–graphitization” sequence, a few other protocols are being developed for GNR synthesis. An example is the Brønsted acid promoted nonoxidative benzannulation of polyalkynylated poly-*para*-phenylene precursors (Scheme 4a).^89^ Another one is the solid-phase topochemical polymerization of diacetylene precursor crystals with subsequent aromatization (Scheme 4b).^90−92^

The essential question of whether such ultralarge carbon nanostructures are structurally perfect and reproducible relates to the methods of synthesis and characterization, both of which are still in need of further improvement. Regarding structure proof, while the whole toolbox of instrumental analysis must be employed, including crystal structure determinations, the macromolecular character and limited solubility of carbon nanostructures are severe obstacles. PAHs larger than HBC revealed such pronounced tendencies for aggregation that solution-NMR spectra could no longer be recorded, which emphasizes the need for various solid-state methods. Further, techniques such as scanning tunneling microscopy (STM) and noncontact atomic force microscopy (nc-AFM) have displayed chemical value in molecular structure visualizations with atomic precision.^30,93−98^ While these tools furnish nice graphics, deposition of molecules on metal surfaces and recording of micrographs might well overlook side products and defects beyond the limited visualization region. On the other hand, failures of graphene syntheses leave defects in the NG- and GNR-structures which, even if minor, may obstruct transport of charge carriers and hamper device performance, but go undetected in spectroscopic or microscopic analyses.

## 3 When Surfaces Come into Play

**Figure 1:**
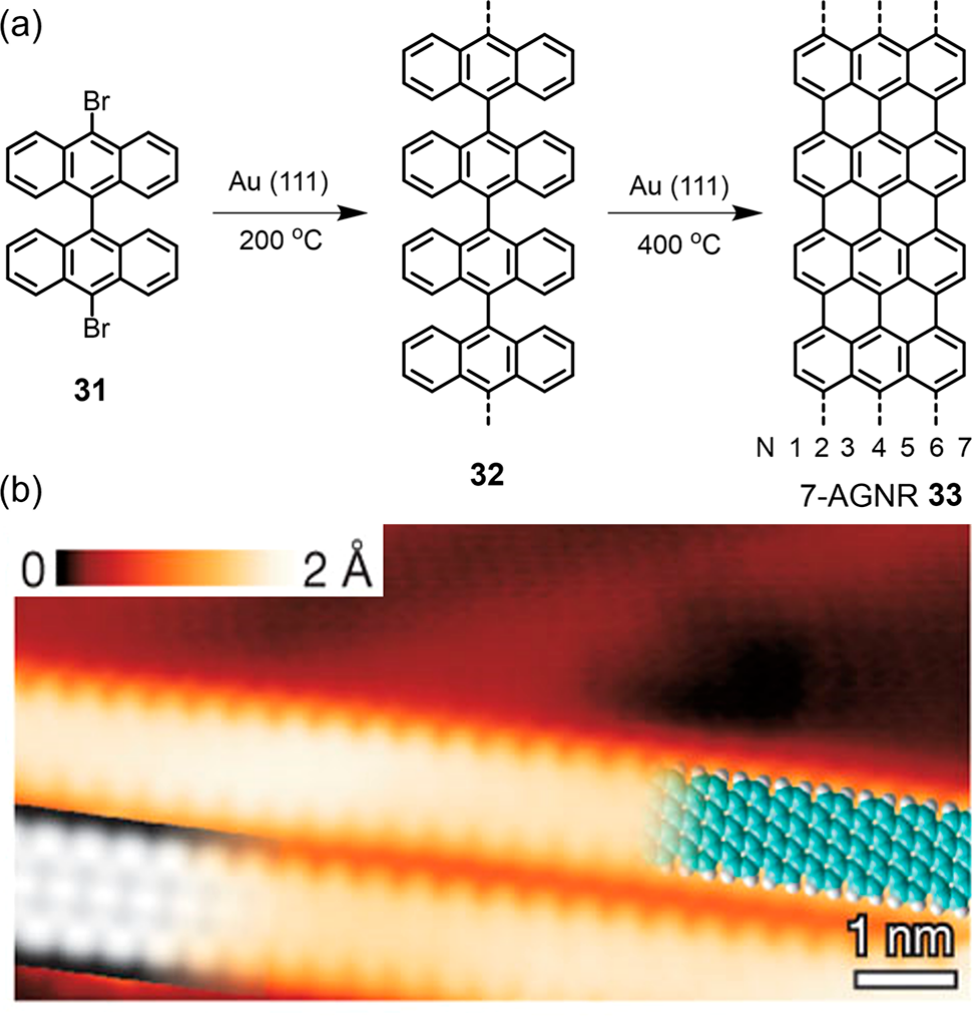
(a) Reaction schemes for 7-AGNR **33**. (b) High-resolution STM image with a partly overlaid molecular model (blue) of **33**. At the bottom left is a DFT-based STM simulation of **33** shown as a grayscale image. Reproduced with permission from ref (95). Copyright 2010 Springer Nature.

**Scheme 5:**
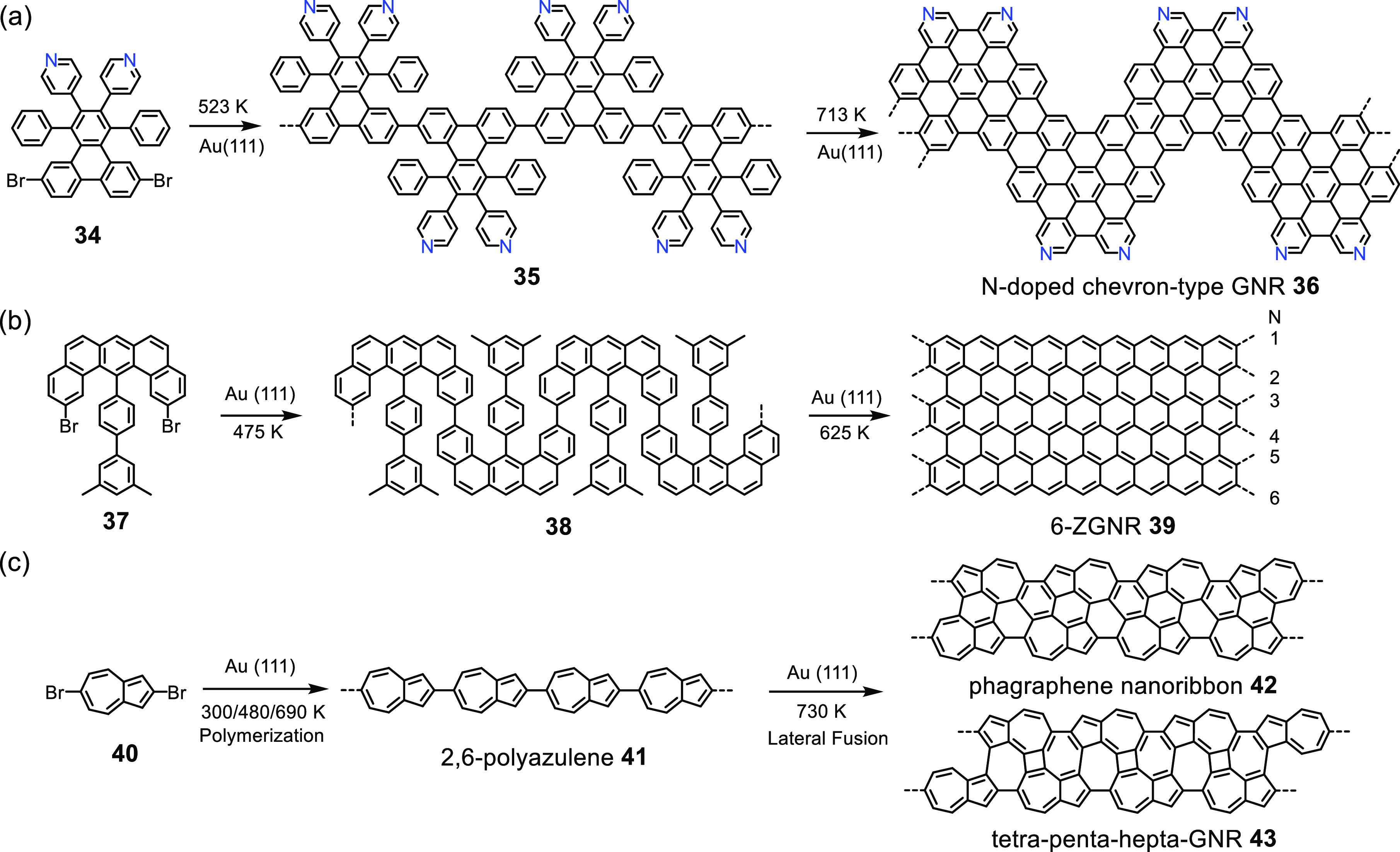
Surface-Assisted Synthesis of (a) Heteroatom-Doped Chevron-Type GNR **36**,^99^ (b) Zigzag-Edged GNR **39**,^98^ and (c) Azulene-Containing GNRs **42** and **43**(103)

In addition to visualizing NGs and GNRs, nanoscience has also played a unique role in synthesis. Thereby, branched oligophenylene monomers equipped with two or more halogen substituents are deposited on metal surfaces by sublimation under ultrahigh vacuum (UHV) conditions. Carbon–halogen bonds are homolytically cleaved upon heating, which furnishes radical species prone to undergo clean polymerization. Further heating can cause cyclodehydrogenations leading to formation of flat graphenic species. The beauty of this approach is that (1) the processes can be monitored *in situ* by microscopies; (2) the radicals are not quenched by the solvent or air under UHV conditions; and (3) complex π-conjugated systems, including those that would not survive in solution, can be made and stabilized by interaction with the metal. The breakthrough in this direction was the synthesis of armchair-edged GNR **33** (7-AGNR, where, again, “7” is the ribbon width) from the dibromobianthryl **31** in our collaboration with group of Roman Fasel more than a decade ago (Figure 1).^95^ Excitingly, the structures of the target GNRs can be designed by the choice of monomer. This holds true for incorporation of heteroatoms or peripheral substituents,^99−101^ the nature of the edges such as the transition from armchair to zigzag peripheries,^98^ and the use of azulene-containing rather than all-benzenoid GNRs.^102,103^Scheme 5 can only provide some typical cases, but at present, the broad scope of this new concept is finding increasing attention.^17,19,41,104−107^ In addition to GNRs, more graphenic structures have been reported from on-surface syntheses, such as porous NGs,^108,109^ nanoporous graphene,^28^ and nonbenzenoid biphenylene networks,^110^ which are difficult or impossible to prepare via traditional solution chemistry.

**Figure 2:**
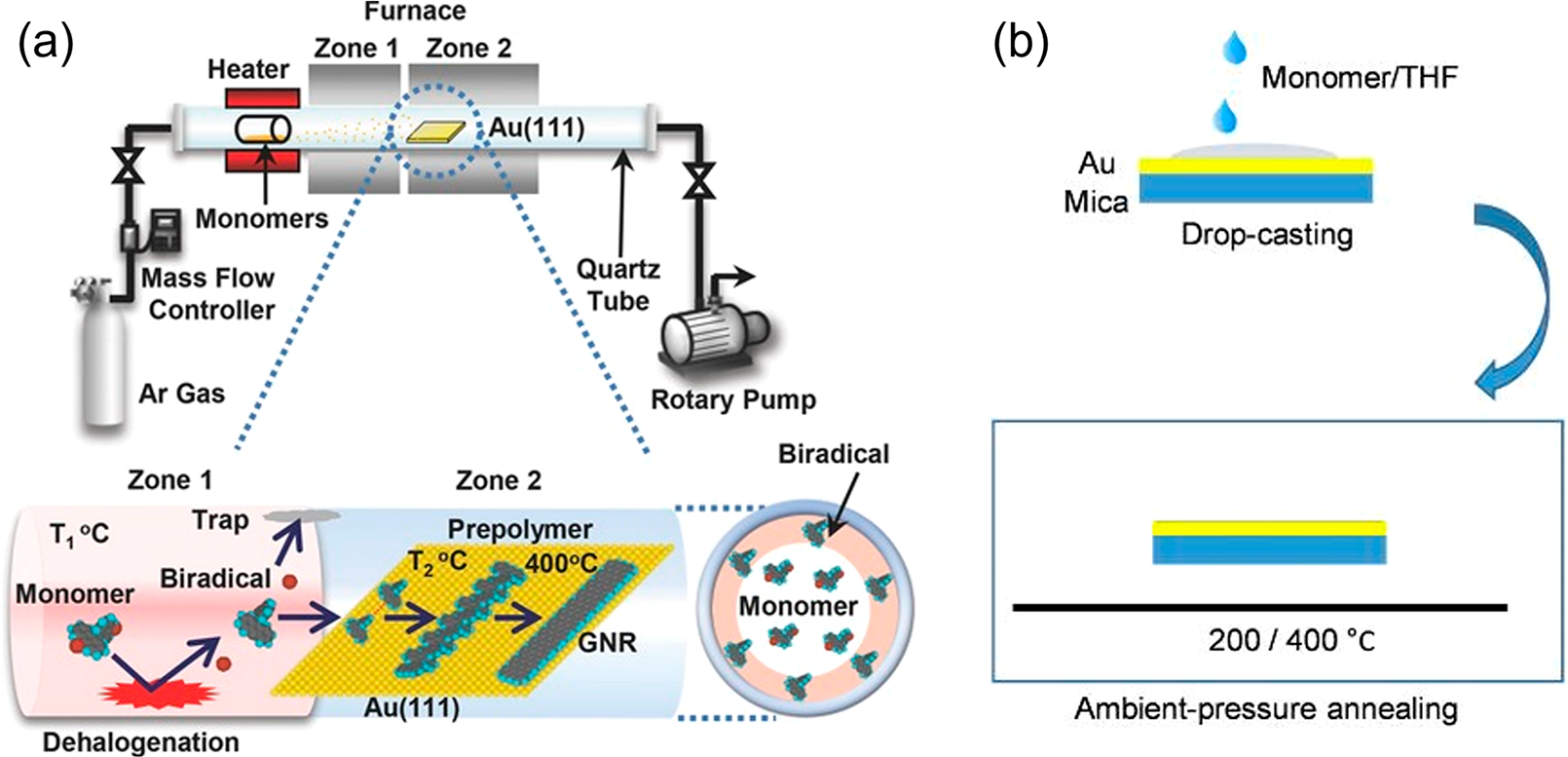
(a) Experimental CVD setup with an illustration of the presumed GNR growth process. Reproduced with permission from ref (112). Copyright 2014 John Wiley and Sons. (b) Schematic illustration of GNR synthesis through solution processing. Reproduced with permission from ref (116). Copyright 2017 The Chemical Society of Japan.

What speaks against this fascinating addition to the toolbox of organic and polymer synthesis is the sophisticated equipment required for surface physics and the extremely small scale, although some upscaling is possible by applying chemical vapor deposition (CVD) under less rigorous conditions.^111−115^ The reaction mechanism for the bottom-up GNR synthesis by CVD is similar to those of the UHV methods, except that trace amounts of oxygen can hardly be excluded in the CVD chamber; this reacts with the diradical intermediates to terminate the polymerization, thus leading to shorter and oxidized GNRs (Figure 2a).^112^ Therefore, it is essential to mix hydrogen with argon in the CVD growth process to suppress oxidation. A recent solution processing method has been developed to produce GNRs by drop-casting monomers on the reaction surface followed by annealing at ambient pressure (Figure 2b).^116^ In addition to the “polymerization–graphitization” protocol, control over GNR structures is realized through *in situ* growth of graphenic materials on certain catalytic templates, including germanium surfaces, hexagonal boron nitride trenches, nickel nanobars/films, and copper twin crystals.^117−120^ Recently, even a template-free CVD synthesis combining liquid copper and controlled etching by hydrogen has been demonstrated as a strategy for tunable growth, large scalability, and fewer defects in GNRs.^121^

Despite the exciting progress in this field, metal-catalyzed on-surface reactions still face many unsolved mechanistic issues: (1) polymerization of diradical intermediates for repetitive CC-bond formation requires migration on the surface, which becomes increasingly difficult for higher oligomers; (2) end-capping by halogen or premature dehydrogenation may quench further reactions; and (3) the nature and surface structure of metals may become decisive, with metal adatoms coming into play as key reagents. Sublimation, even under UHV conditions, is limited by molecular size. In cases of ultralarge molecules, laser-supported deposition combined with soft-landing methods or deposition by vapor-phase transport has pushed the limits of processing in the gas phase.^122,123^ Further, control of on-surface GNR synthesis might comprise (1) the choice of the halogen substituent with different initiation temperatures; (2) the regiochemistry of asymmetric dihalo oligophenylenes; or (3) simultaneous introduction of different monomers, e.g., with electron-donating and electron-withdrawing substituents, which might furnish molecularly defined p–n junctions. Unlike solution synthesis, this on-surface chemistry is not troubled by solubility issues, but further applications of the GNRs in electronic devices will require lift-off from the surface and transfer to insulating substrates, either by etching of the metal surface or electrochemical delamination of the GNR films.^104,112,113,124−126^ These protocols, apart from necessitating a costly extra processing step, are still in need of further improvement. Therefore, establishing reliable on-surface synthetic methods directly on insulating substrates is critically important toward, for example, (opto)electronic and spintronic applications.^127−130^

## 4 Rising from Flatland

Using the branched polyphenylenes as carbon reservoirs, 3D precursors have been planarized to the graphenic “flatland”.^131^ Thereby, the topologies of the precursors in section 2 are designed to avoid spatial overlap of benzene rings during flattening. While this is crucial for accessing planar molecular nanocarbons, the cyclodehydrogenation reaction has also been successfully applied in the syntheses of nonplanar molecular structures despite the existing strain.^132−139^ By incorporating nonhexagonal rings into the “honeycomb” framework, curved NGs are obtained with bowl-shaped or saddle-shaped surfaces.^140−144^ In addition to the Scholl reaction, ring expansion, cyclotrimerization, intramolecular Friedel–Craft cyclization, Pd-catalyzed C–H arylation, and cascade radical photocyclization are also used to construct five-, seven-, or eight-membered rings.^144−148^ New opportunities for optical and electronic properties, especially chirality-related characteristics, have been demonstrated in various curved NGs with out-of-plane deformation of π-conjugation.^137,149,150^ The negatively curved NGs have the potential to self-assemble in organic solvents and serve as efficient gelators.^133^ The studies of curved NGs can also stimulate bottom-up syntheses and characterization of 3D carbon nanostructures,^151^ such as fullerenes,^152−155^ carbon schwarzites,^140,156^ Mackay crystals,^157,158^ and carbon nanosolenoids with Riemann surfaces.^159^ In a similar fashion, NGs can be twisted and bent by constructing the aliphatic chains as intramolecular bridges as in, for example, cyclophanes.^160^

**Figure 3:**
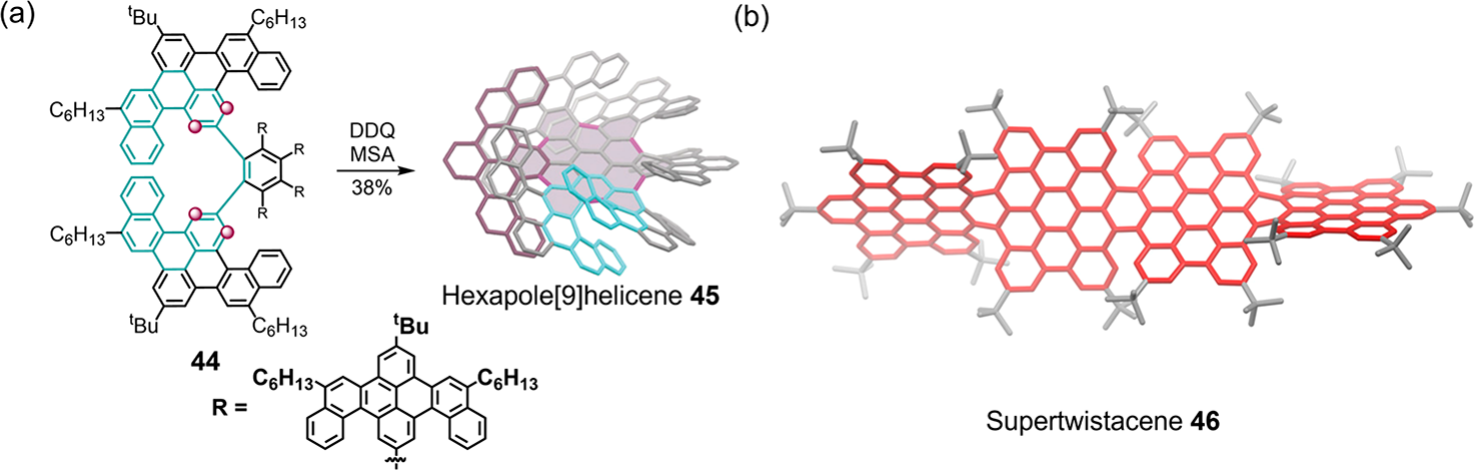
Molecular models of NGs with multiple helical edges: (a) Synthesis of hexapole[9]helicene **45** from **44** via Scholl Reaction (reaction positions are highlighted with purple circles). Reproduced with permission from ref (161). Copyright 2018 John Wiley and Sons. (b) Supertwistacene **46**. Reproduced with permission from ref (135). Copyright 2020 American Chemical Society.

When “rising from the flatland”, chirality and handedness are no doubt the holy grail of synthesis. The resulting chiroptical features, such as circular dichroism and circularly polarized luminescence, are fascinating and have been studied for circularly polarized organic light-emitting diodes and chiral bioimaging applications.^162,163^ Chiral molecules not only interact with photons to produce chiroptical signals but also influence the spins of the electrons passing through the structures.^164^ This phenomenon, known as the chiral-induced spin-selectivity effect, has many potential applications in, for example, biorecognition as well as spintronics.^165−167^ Combinations of NGs and helicenes are therefore attracting increasing attention, and NGs can provide high hole mobility and an extra platform for chemical modifications.^168−175^ The chemistry of NGs, especially the cyclodehydrogenation reaction, has played a key role in substantially extending the π-conjugated systems of helicenes. This has provided multiple helical edges in NGs, such as hexapole [9]helicene **45** and supertwistacene **46** (Figure 3).^135,161^ The Scholl reaction can also deliver helical structures regioselectively from naphthalene, phenanthrene, furan, and thiadiazole building blocks^137,176−178^ in which the position with higher electron density appears to favor cyclodehydrogenation. Besides the Scholl reaction, the transition-metal-catalyzed [2 + 2 + 2] cycloaddition is another powerful tool to synthesize helical NGs.^179,180^ The π-extended helicenes, sometimes referred to as superhelicenes, possess intriguing mechanical, electronic, magnetic, and spin properties as nanosprings^181^ and nanosolenoids.^149^ In addition to the neutral π-extended helicenes, the charged species obtained through metal reduction offer new possibilities to engineer the geometry, aromaticity, and electronic structures.^182−185^

**Figure 4:**
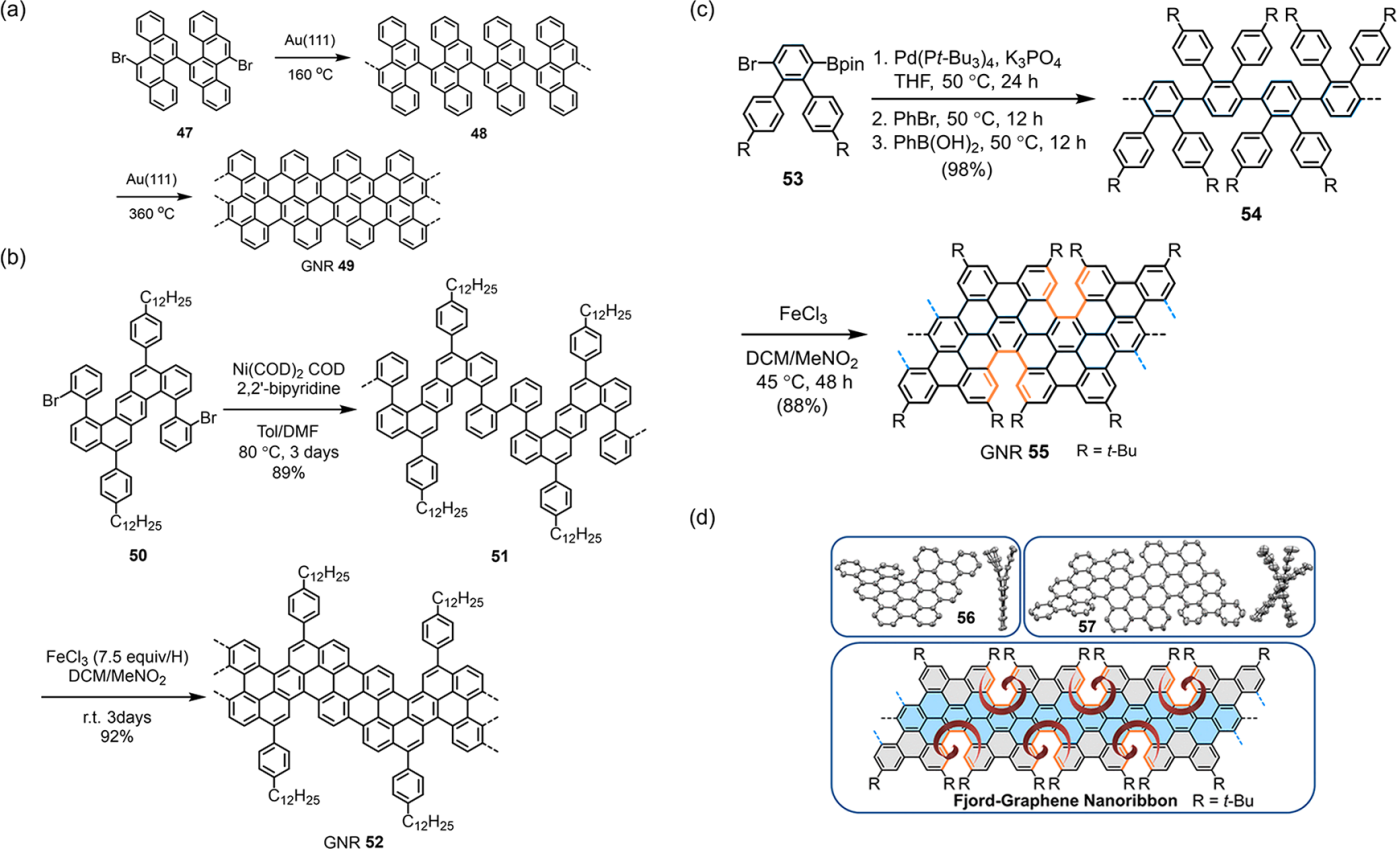
Synthetic routes toward (a–b) Cove-edged GNRs **49**, **52** and (c) Fjord-edged GNR **55**. (d) X-ray crystallographic analyses of fjord-edged model compounds **56** and **57**, as well as geometrical envisioning of the corresponding GNRs **55**. Reproduced with permission from ref (26). Copyright 2021 American Chemical Society.

“Superhelicenes” are expected to be superior to conventional helicenes in view of their enhanced chiroptical and electronic properties. A further extension from helical NGs to helical GNRs is expected to provide amplified chirality and electron conductivity due to the polymeric nature of GNRs. Precise structural control of nonplanar GNRs, including their size, length, edge structure, and handedness, is expected to be more demanding than that of their small molecular analogs, partly due to the strain accumulated along the polymer backbone. A smart strategy is to utilize the steric hindrance on the edge to create nonplanarity only on the periphery, as demonstrated by the cove-^18,24^ and fjord-edge^26^ GNRs reported recently (Figure 4a–c). While the nonplanar edge structures can be disclosed by X-ray crystallographic analyses of the model compounds **56** and **57**, the corresponding GNRs **55** are only tentatively envisioned to possess a single site of chirality on the edges (only *M* or only *P*) in the most stable geometry (Figure 4d).

**Figure 5:**
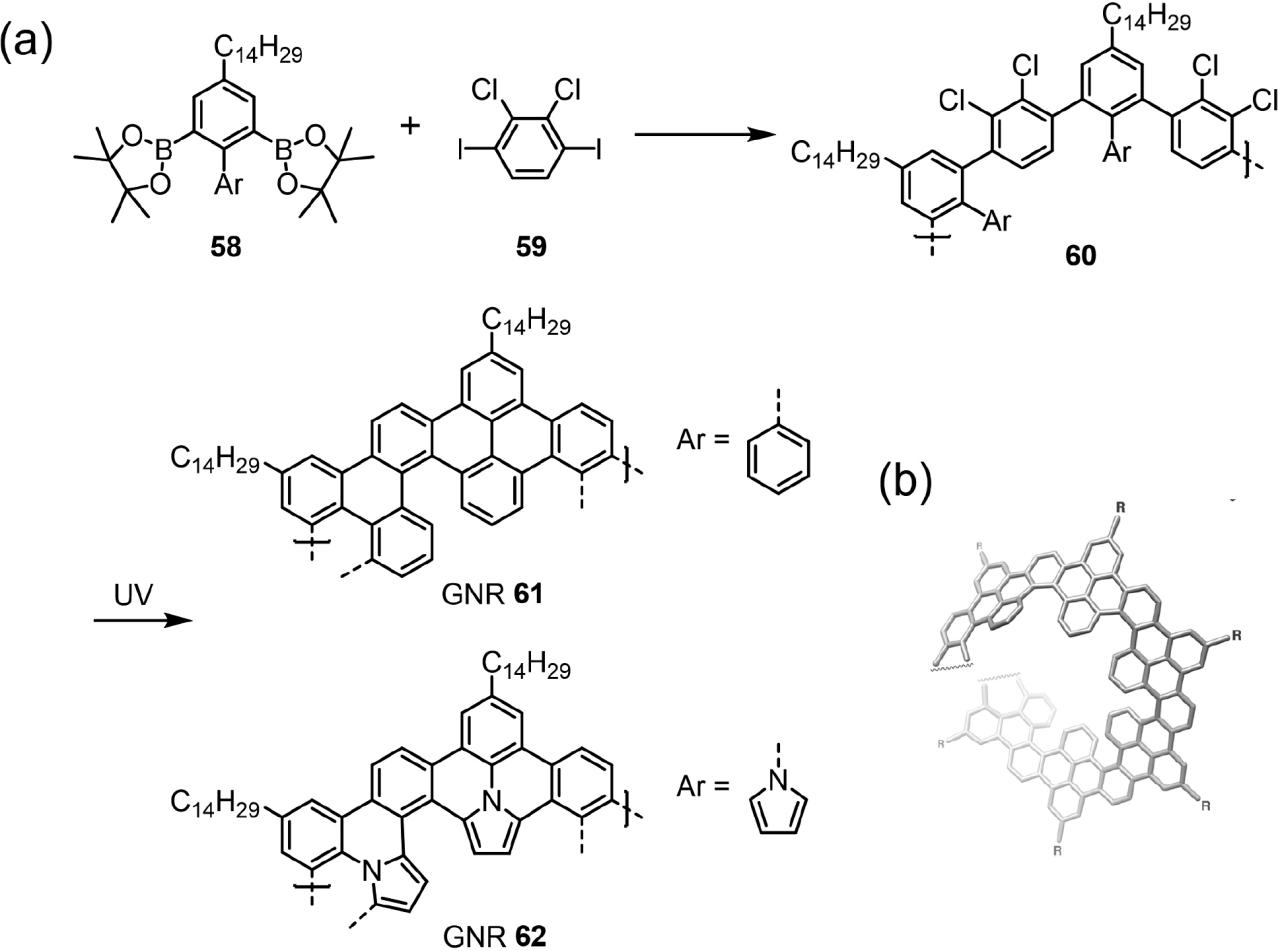
(a) Helically coiled GNRs **61** and **62** from Suzuki polymerization followed by a photochemical cyclodehydrochlorination reaction. (b) Helical structure of GNR **61** simulated by DFT calculations.

Such multihelicity on the edge together with the intrinsic polydisperse nature of the GNRs will unavoidably prevent chiral separation and investigations of their chiral properties. The helicene-like GNRs **61** and **62** synthesized by the photochemical cyclodehydrochlorination of the chlorinated polyphenylene precursors provide promising examples of chiral GNRs with single-handedness (Figure 5).^22,25^ Unfortunately, the helicity is created during the cyclodehydrochlorination without chiral selectivity, meaning that the obtained GNRs are still racemic mixtures. There is, thus, plenty of room to adopt state-of-the-art asymmetric syntheses to fully unleash the potential of chiral GNRs.^186^

**Figure 6:**
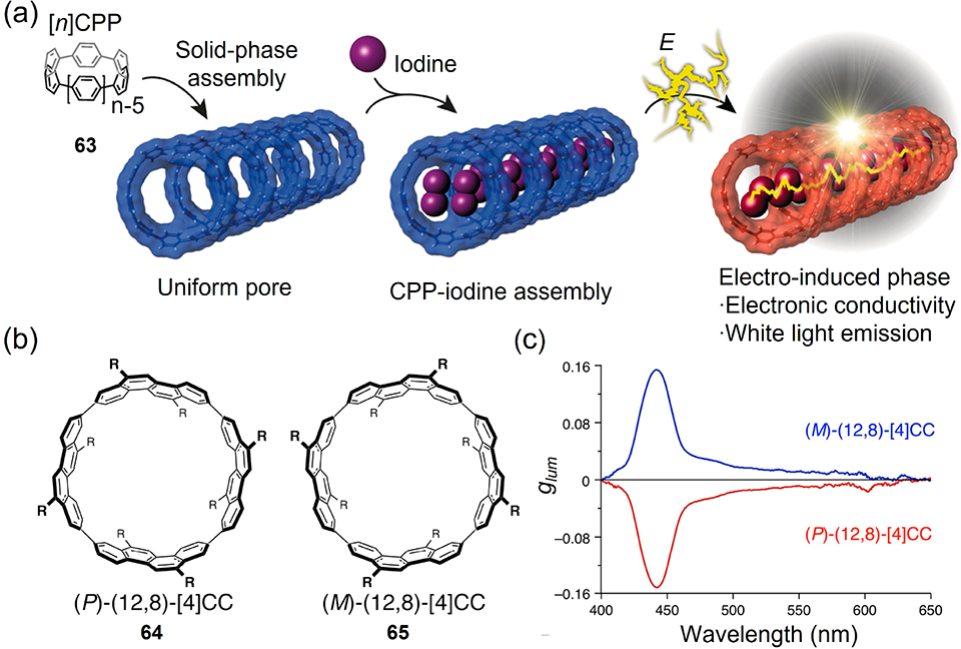
(a) Electric-stimulus-induced phase transition of the [10]cycloparaphenylene-iodine complex and turn-on electrical conductivity as well as white light emission. Reproduced with permission from ref (197). Copyright 2017 John Wiley and Sons. (b) Structure of (*P*)- and (*M*)-(12,8)-[4]cyclo-2,8-chrysenylene. (c) Circularly polarized luminescence spectra of **64** and **65** in toluene solution. Reproduced with permission from ref (198). Copyright 2017 National Academy of Sciences.

In addition to the helical carbon structures, carbon nanorings and nanobelts, which are regarded as the molecular subunits of CNTs, are related cases that comprise closed loops of polyphenylenes and fully fused benzene rings, respectively.^86,187−191^ Their optoelectronic properties can be engineered by modifying the ring size, width, and edge structures.^191,192^ Chirality can also be found in carbon nanorings and nanobelts due to the absence of an inversion center and symmetry plane.^193^ Even more complicated ring-shaped carbon species are highly twisted macrocycles (figure-eight, Moebius ring, etc.) and trefoil knots.^194−196^ These nanocarbons have attracted chemists for decades, not only because of their appeal as synthetic showcases but also due to their unique properties and applications in supramolecular chemistry and optoelectronics.^191,192^ For instance, the crystalline [10]cycloparaphenylene–iodine complex furnished electrical stimuli-responsive white light emission and “turn on” electrical conductivity (Figure 6a).^197^ Another convincing example is the highest organic luminescence dissymmetry factor (*g*_lum_ = 0.152) obtained from a tubular molecule, (12,8)-[4]cyclo-2,8-chrysenylene **64** and **65** ((12,8)-[4]CC) in Figure 6b–c) with an emission quantum yield of 80%.^198^ Using theoretical calculations, the dissymmetry factor was found to benefit directly from the large magnetic dipole transition moment parallel to its unique cylinder topology.

**Figure 7:**
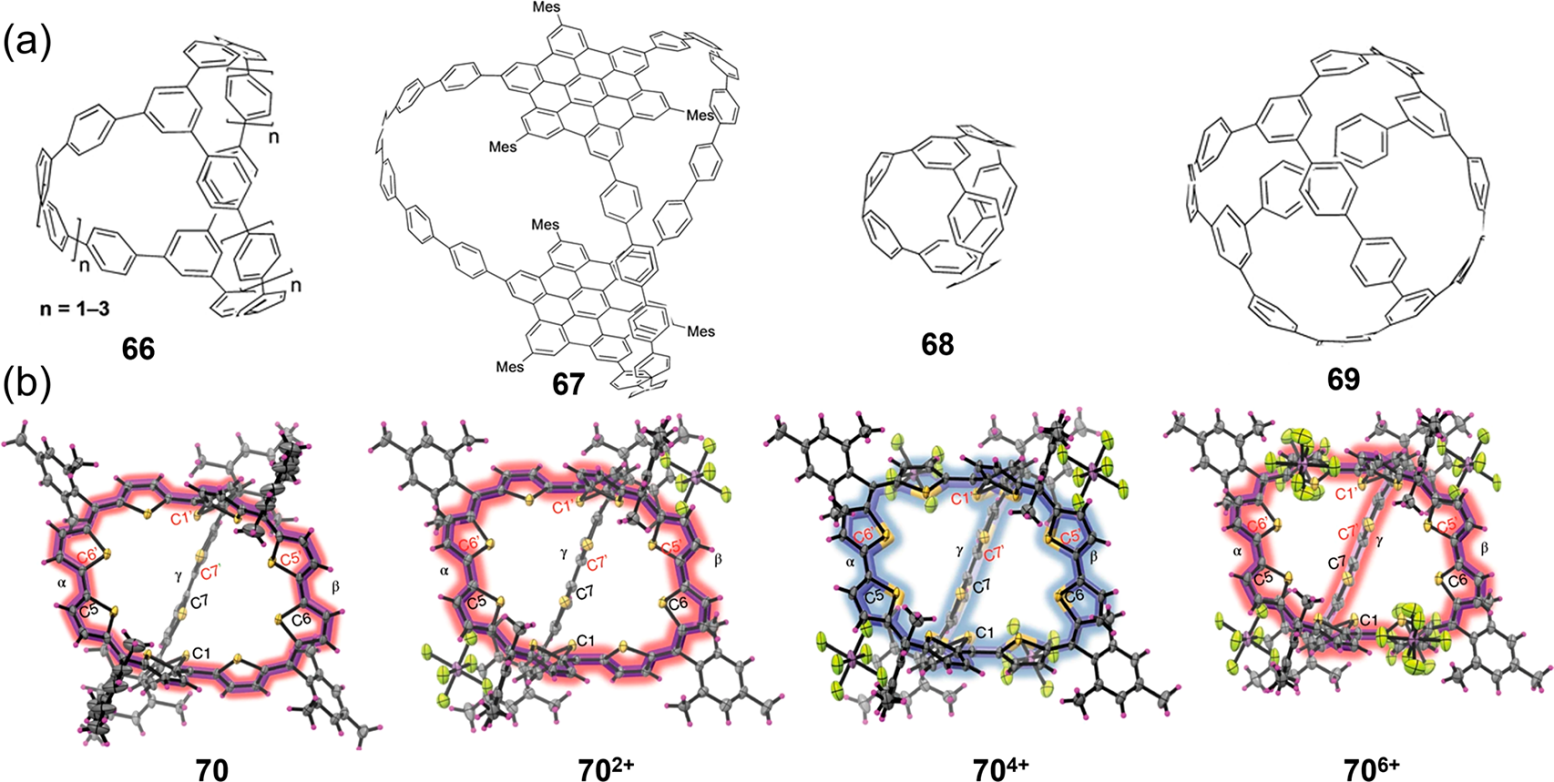
(a) Spherical carbon nanocages **66**–**69**. (b) X-ray crystallographic structures of the threefold symmetrical and fully conjugated molecular cage **70** with different oxidation states. Reproduced with permission from ref (199). Copyright 2020 Springer Nature.

When multiple rings are interconnected, 3D hydrocarbon-based molecular cages and nanotubes can be constructed with permanent holes, which allow applications in host–guest chemistry, chemical sensing, and gas adsorption/separation.^200−202^ The “perfect” example is, of course, the famous fullerene C_60_, while there are many more hydrocarbon cages that have been synthesized by various carbon–carbon coupling reactions (Figure 7a).^203−208^ Using benzene as the repeating unit, the aromaticity in 3D π-conjugated molecules appears to be a challenging topic. One notable example is Hirsch’s 2(*n*+1)^2^ spherical aromaticity rule for fullerenes, where π-electrons are delocalized through the whole spherical framework.^209,210^ A recent example is the *C*_3_ symmetrical and fully conjugated molecular cage **70** synthesized by connecting two carbon-centered radicals with three identical conjugated bridges (Figure 7b). Different types of aromaticity (Hückel, Baird, and 3D global aromaticity) occur in this cage with different oxidation states.^199^

## 5 Access to High-Spin Materials

The characteristic structural features of GNRs, which have been introduced above, are all relevant for the electronic band structure of GNRs and, in turn, offer enormous opportunities for fine-tuning their photophysical and optoelectronic properties. This has been extensively described in other review articles.^17,19,38,105^ Herein, another important method for electronic structure control is emphasized, that is, bringing spins to molecular graphenic structures. Controlling quantum degrees of freedom is an important challenge of physics, and “quantum matter” has been realized in, for example, ion traps or arrays of cold atoms, but often the underlying coupling energies remain low. Attention has therefore been directed to π-radicals as constituents of highly entangled spin chains or superlattices with strong interactions that originate from unpaired π-electrons or partially filled π-bands.^33,98,211^

**Figure 8:**
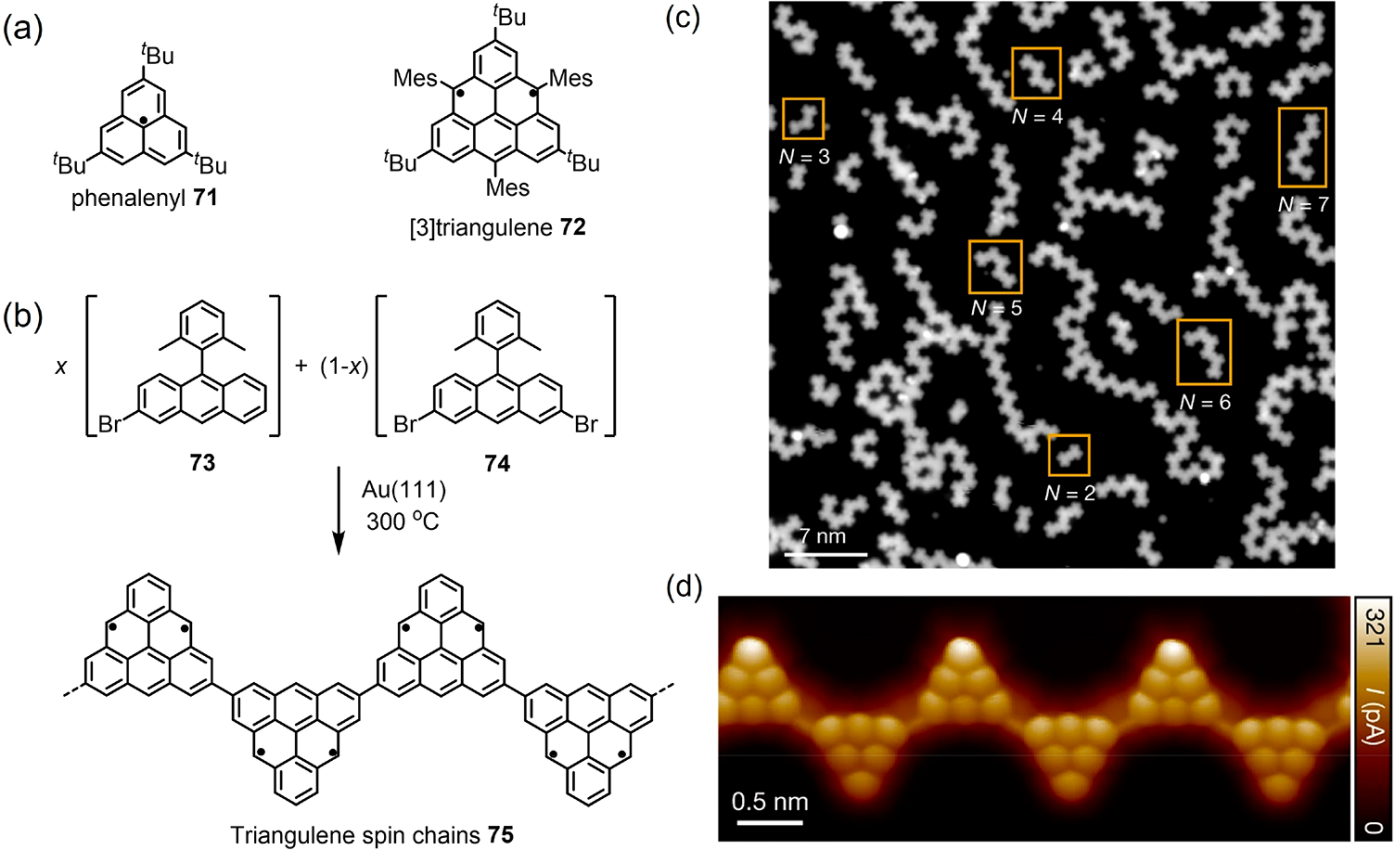
(a) Structures of phenalenyl **71** and [3]triangulene **72**. (b) On-surface synthesis of triangulenyl spin chains using a precursor mixture of **73** + **74**. (c) Overview STM image after annealing the precursor mixture (*x* = 0.2) on Au(111) at 300 °C. (d) Bond-resolved STM images of triangulenyl spin chains **75**. Reproduced with permission from ref (212). Copyright 2021 Springer Nature.

Such building blocks are, for example, the phenalenyl **71** and [3]triangulenyl **72** systems (Figure 8a), which can be kinetically stabilized in the crystalline state by introducing bulky groups (*tert*-butyl or mesityl substituents).^213,214^ Compared to solution-mediated syntheses, these radicals can be synthesized and made more persistent by thermal CH-cleavage from closed-shell precursors on metal surfaces. This concept can go even further since the triangulenyl lattice has been made via metal catalysis by ring closure from 9-(2,6-dimethylphenyl)anthracene and the triangulenyls assembled into networks when using halogen-substituted derivatives (Figure 8b–d).^212,215^

**Figure 9:**
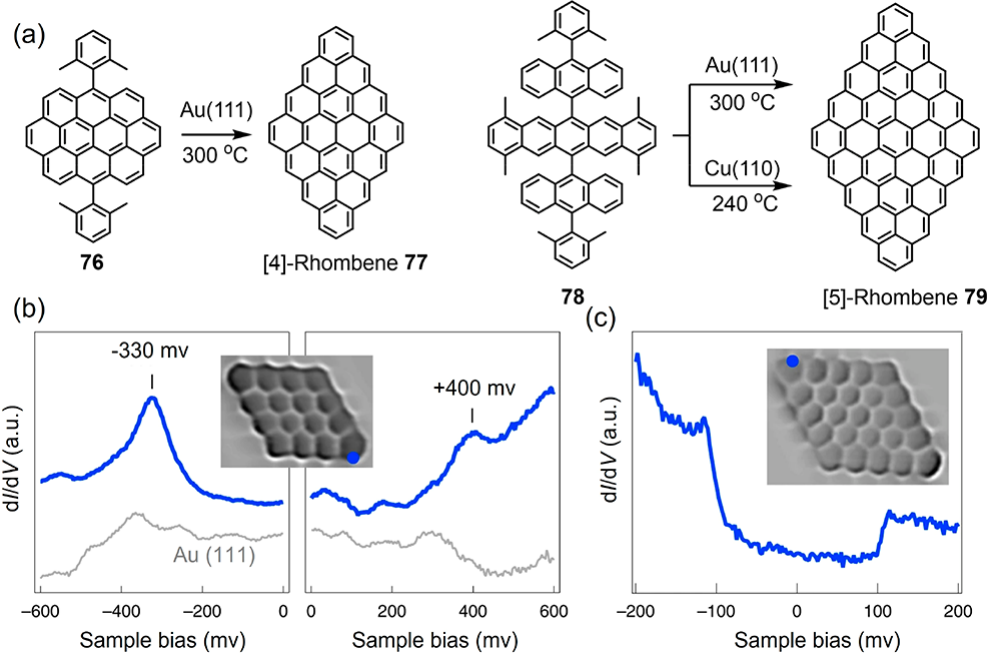
(a) Synthetic schemes for [4]- and [5]-rhombenes. (b) d*I*/d*V* spectra of **77** with HOMO and LUMO resonances at −330 mV and +400 mV, respectively. (c) Background-subtracted d*I*/d*V* spectrum of **79** revealing inelastic excitation steps. Reproduced with permission from ref (216). Copyright 2021 Springer Nature.

Another way to access high-spin structures and nontrivial magnetism is the design of zigzag-edges as occurring in the new rhombus-shaped discs [4]- and [5]-rhombene (**77** and **79**, Figure 9).^216^ Scanning tunneling spectroscopy (STS) analysis of [4]- and [5]-rhombene revealed an emergent magnetic spin-singlet ground state with increasing nanographene size, and a magnetic exchange coupling of more than 100 meV, significantly exceeding the room temperature Landauer limit.

**Figure 10:**
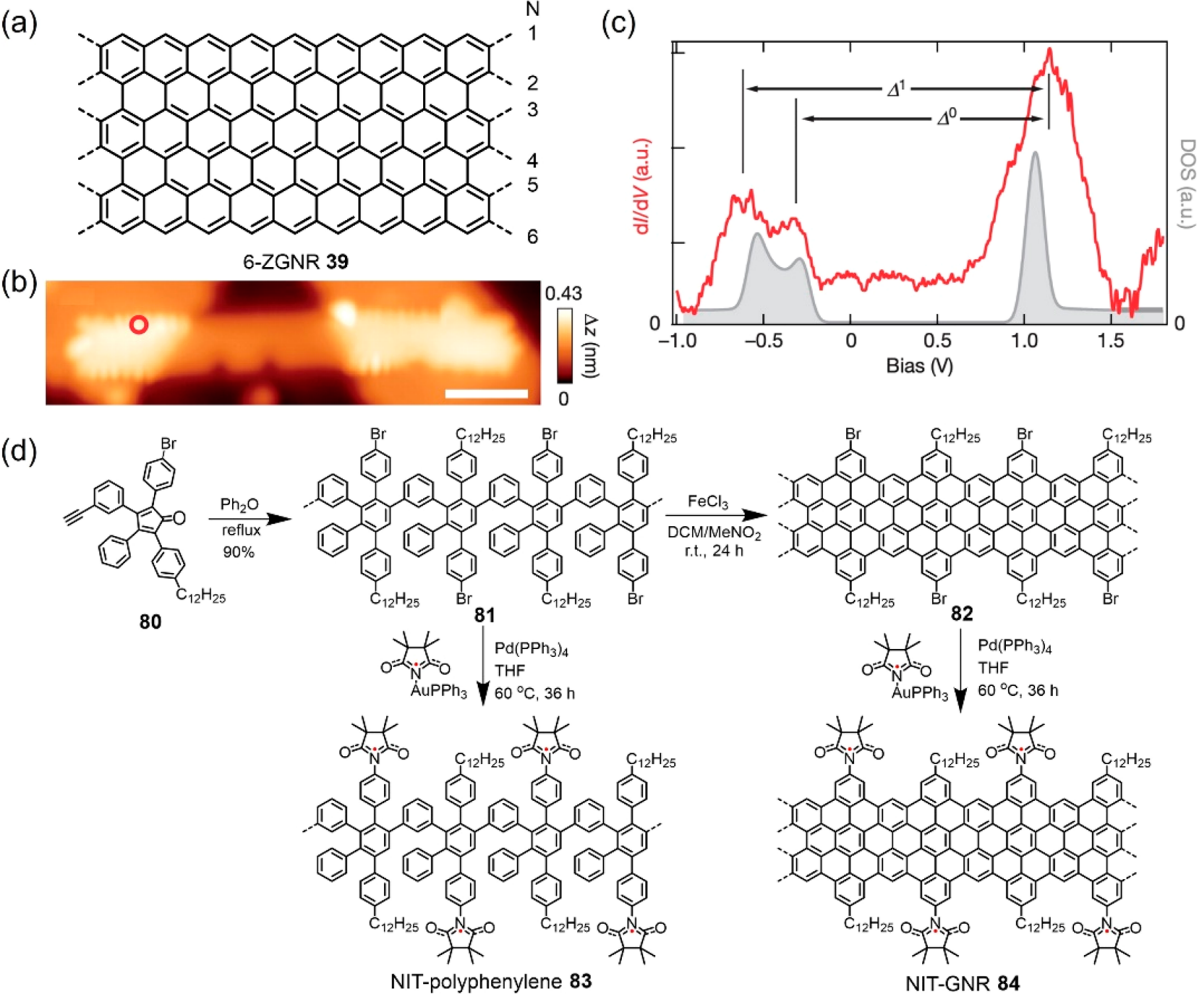
(a) Structure of 6-ZGNR **39**. (b) STM topography image of 6-ZGNR **39** bridging between two NaCl monolayer islands. (c) Differential conductance (d*I*/d*V*) spectrum (red) taken at the zigzag edge marked by the red circle in (b), and the quasiparticle density of states (DOS; gray). Reproduced with permission from ref (98). Copyright 2016 Springer Nature. (d) Synthetic route to NIT-GNR **84** decorated with nitronyl nitroxide (NIT) radicals at the periphery.

Extending this concept to GNRs has prompted the synthesis of zigzag-edged GNR **39** (6-ZGNR, where “6” is the ribbon width as above), which was shown by density functional theory (DFT) calculations to possess extremely low band gaps as well as edge-localized electronic states with energy splittings.^98^ These features lead to magnetism and spin-filtering behavior (Figure 10).^98,217−219^ Without doubt, magnetic edges in GNRs and controlled manipulation of them would define a breakthrough for spintronics and quantum computing. On-surface syntheses of such GNRs, as sketched within Scheme 5b in Section 3, imply CC-bond formation by methyl-aryl coupling next to electrocyclic ring closure by aryl–aryl coupling, which highlights the importance of precursor design. The synthesis of the U-shaped precursor dibenzo[*a*,*j*]anthracene **37** with additional phenyl and/or methyl substituents is a convincing case of GNR-edge control.^220^ The chemical instability of ZGNRs and the importance of spins delocalized over GNRs have suggested yet another approach depicted in Figure 10d.^221^ There, stable GNRs **84** are synthesized and their peripheries are decorated with persistent radicals that can partially delocalize spin density onto the GNR. Characterization of the resulting conjugates by electron spin resonance (ESR) spectroscopy will be mentioned in section 7.

**Figure 11:**
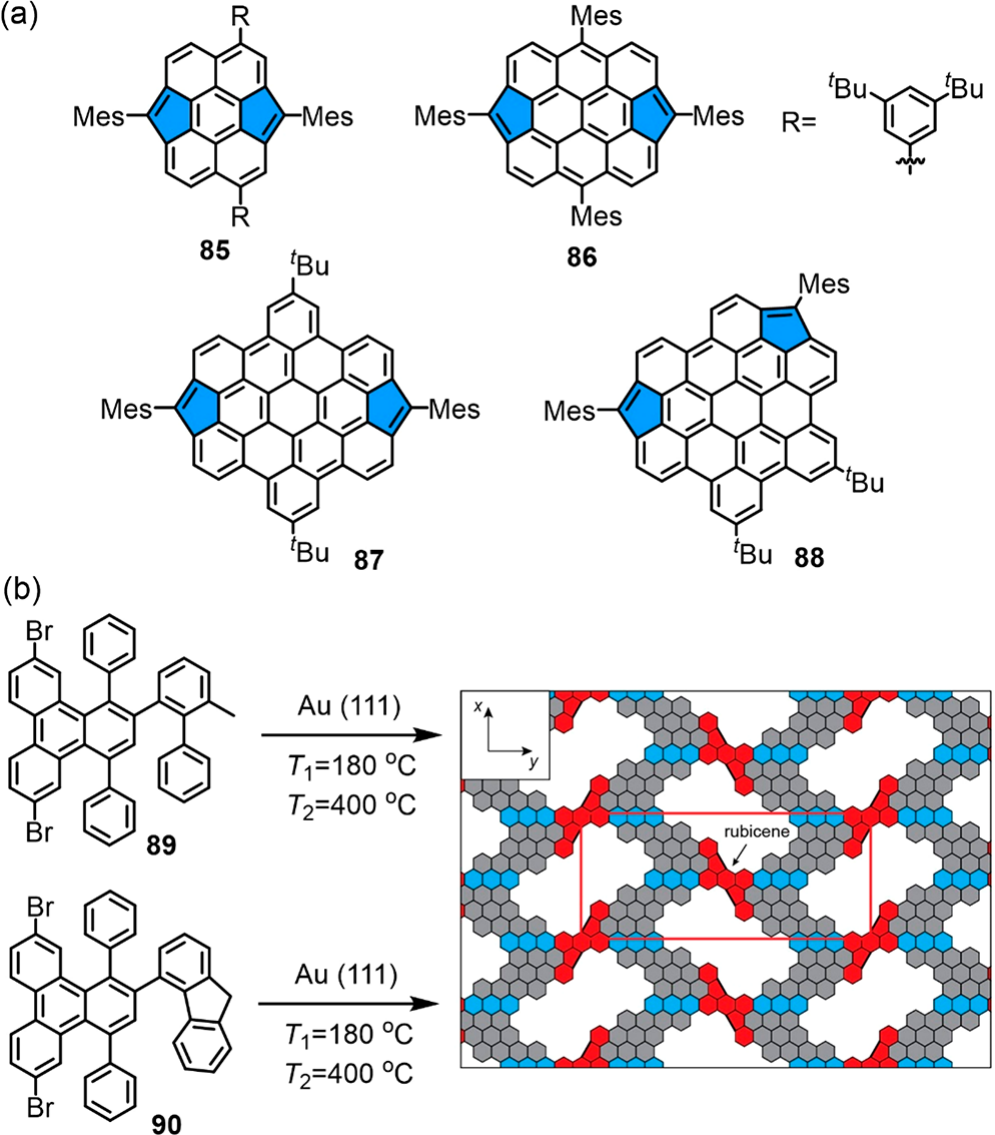
(a) Structures of bis-pentagon derivatives of perylene **85**, bisanthene **86**, and HBC **87**–**88**. (b) On-surface synthesis of a pentagon structure through cyclization of polyphenylenes **89** and **90** with methyl and methylene groups. Reproduced with permission from ref (222). Copyright 2020 American Chemical Society.

Another concept with relevance for quantum applications is formation of spins in a semiconductor when starting with defects.^27,223−225^ One possibility is to enclose five- or seven-membered rings in the hexagonal matrix.^27,226,227^ In graphene, dislocations giving rise to two pentagon–heptagon pairs, known as a Stone–Wales defect,^228^ can still migrate around, whereas the present molecularly defined graphene chemistry can provide static and carefully engineered defects. While azulene-based GNRs have been mentioned above, there is much to learn from NGs and defined oligomers. Recent cases are the bis-pentagon derivatives of perylene **85**,^229^ bisanthene **86**,^230^ and HBC **87**, **88**(231) (Figure 11a). A comparison of the *para*- or *meta*-fused biradicals in HBC (**87** and **88** in Figure 11a, respectively), which are formed by oxidation of the corresponding dianions, is revealing. When attempting to describe the bonding situation in NGs by closed-shell Kekulé structures, the number of six-membered rings maintaining their resonance stabilization is the decisive factor, and a singlet-ground state structure of **87** would cost too many benzene resonance energies. Pentagon structures are also accessible in on-surface protocols when a methyl group is placed inside a bay region, as in the case of polyphenylenes and GNRs.^232^ The substituted polyphenylenes **89** and **90** containing methyl and methylene groups yield chevron-type GNRs that are further fused into nanoporous graphene on the surface (Figure 11b).^222^

## 6 Size Matters

The slogan “size matters” is commonly put forward in advertising the chemistry of nanoparticles such as, for example, polymer latex particles or inorganic quantum dots. Critical features are the surface-to-volume ratio or quantum confinement effects. However, size also matters when one proceeds from PAHs to increasingly larger NGs, which leads to bathochromic shifts of optical absorption bands.^64^ Tailoring the HOMO–LUMO energy gaps of NGs not only by size but also by edge and topology^233−235^ provides access to near-infrared absorbers, in particular, in conjunction with attached auxochromic carboxamide groups. The latter are of enormous technical importance in diverse areas such as laser welding, security printing, photodynamic, and photothermal tumor therapies.^236−240^

**Figure 12:**
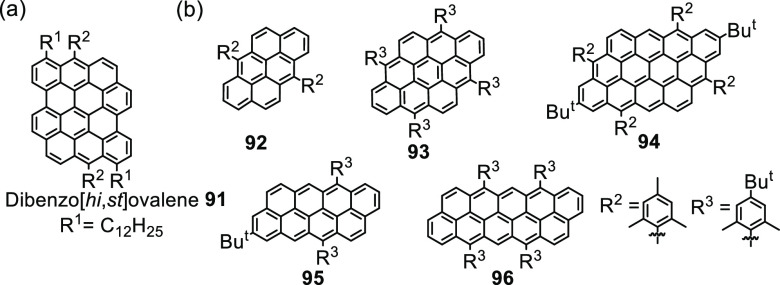
Representative NGs displaying stimulated emission: (a) Dibenzo[*hi, st*]ovalene **91**. (b) *Peri*-acenoacenes **92**–**96**.

The optical properties of GNRs stand in sharp contrast to those of graphene. Whereas visible light is absorbed by graphene independent of the wavelength, absorption by GNRs varies with the width and edge structure of ribbons that can be tuned chemically.^23,241,242^ Even more exciting aspects are the dynamics of their excited states. While recombination of excitons leads to single-photon emission from GNRs,^243^ stimulated emission resulting from biexcitons has also been reported for GNRs under high-excitation conditions, thus opening up opportunities in lasing applications.^32^ In addition, NGs with zigzag edges such as the dibenzo[*hi, st*]ovalene (DBOV, **91**) and *peri*-acenoacenes **92**–**96** help to discover the underlying dynamics of stimulated emission, and one may well envisage opportunities for light amplification in tunable lasers or light-emitting diodes (Figure 12).^244−248^

**Figure 13:**
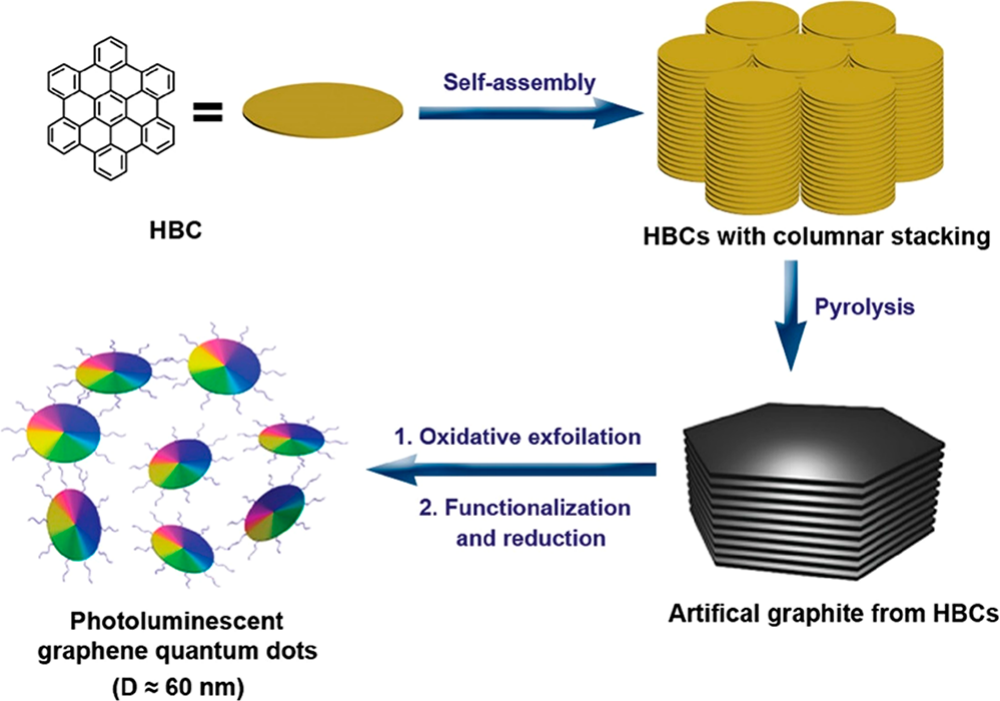
Processing diagram for the preparation of photoluminescent GQDs by using HBC as the carbon source. Reproduced with permission from ref (249). Copyright 2011 American Chemical Society.

The molecule and particle worlds are closely connected in the case of so-called graphene quantum dots (GQDs), which contain graphene sheets within the nanometer length scale and exhibit quantum confinement as well as edge effects.^250^ While several strategies have been employed in making GQDs, such as solvothermal routes,^251^ opening of fullerene cages,^252^ and modern lithography techniques,^253^ synthesis of monodisperse GQDs with defined morphology is still a critical issue. NGs are, in a sense, molecularly defined GQDs but can also be used as a source for bottom-up GQD-fabrication by a sequence involving columnar stacking, pyrolysis, and exfoliation (Figure 13).^249,254^

As was mentioned above, formation of monolayers or thin films is a major prerequisite in many nanoscience experiments and in device fabrication. Processing in solution mostly requires attachment of alkyl chains to the aromatic cores, in particular branched ones, which enhance solubility due to increased solution entropy in organic solvents. This will also result in nanophase separation between the “hard” aromatic disc and the “soft” aliphatic periphery and promote the formation of mesophases.^255−257^ Liquid-crystalline phases have been considered for improving charge-carrier transport. While the hexaalkoxy triphenylenes possess an extremely narrow mesophase width, the discotic mesophase of hexaalkyl HBCs can persist in a wide temperature range from 110 to 420 °C, which offers much better opportunities for device fabrication.^258−260^ The size, shape, and periphery of discotics are decisive. This is easily understood by considering that the preferred angle of twist in a columnar stack is not necessarily the one delivering the strongest electronic coupling between the layers.

There are many more similar size effects of graphenic layers at different levels of technology,^45,129,261−263^ such as in attempts at minimizing friction.^264^ Lubrication with graphite or 2D materials is well-known, but only precise GNRs offer defined nanocontacts on substrates.^265^ GNRs have been shifted around on gold surfaces by an AFM tip with a nearly superlubric motion, and one relevant feature is, again, the size of the ribbons.^265^

## 7 Changing the World of Electronics

**Figure 14:**
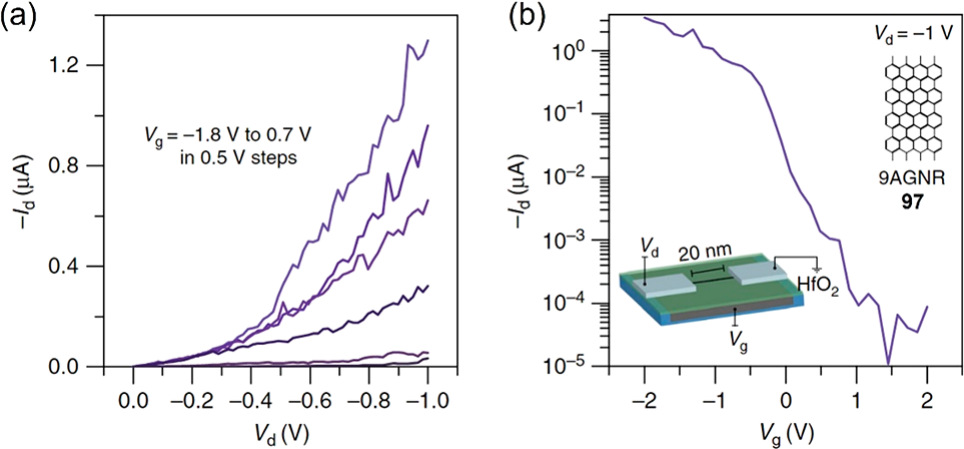
(a) *I*_d_–*V*_d_ and (b) *I*_d_–*V*_g_ characteristics of the untrascaled, high-performance 9-AGNR **97** FET at room temperature. Adapted with permission from ref (266). Copyright 2017 Springer Nature, http://creativecommons.org/licenses/by/4.0/.

In seeking an FET with appropriate performance and switching behavior, GNRs with sufficient length and narrow width are needed to achieve a low band gap. Moreover, the mobility of charge-carrier transport, a decisive process of an FET, is often sensitively dependent on the degree of order in semiconducting materials. To improve device performance, an FET can be built from low-band gap UHV-grown 9-AGNR **97** which is transferred from gold to a hafnium oxide gate dielectric. In sharp contrast to the low ratios observed for graphene, a high on/off ratio of 10^5^ has been achieved from this GNR-based FET device (Figure 14).^266^ Casting networks from solution-made GNRs are much easier and more robust, but the achievable mobilities are largely determined by interribbon transport.^267−270^ In a single-ribbon experiment, however, the current is mainly limited by tunneling through the Schottky barrier at the contact with the electrodes.^37,113,266,271^ To reduce this contact resistance, the use of graphene electrodes proves to be an effective strategy.^272^ A comparison of FETs using gapless graphene and GNRs was provided in a recent review.^104,273^

When proceeding toward other applications, a closer look at theoretical work is appropriate. As expected, changes in band gaps as a function of length, width, and edge structure have stood in the foreground, but additional features have come into play as well, such as the role of end states, noncontinuous changes of properties with length, and the possible influence of a metal substrate on band gaps.^15,23,31,45^ In the literature, experimental and theoretical values of GNR band gaps differ widely, which is partly due to the lack of information on methods of synthesis and thus structural perfection or unspecified length.^15,23^

**Figure 15:**
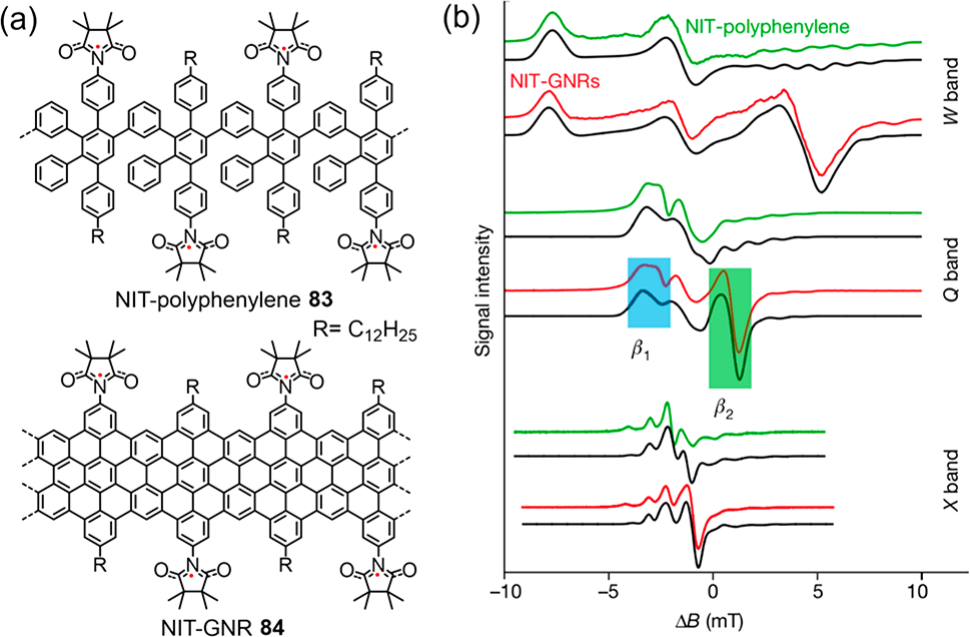
(a) Structures of NIT-polyphenylene **83** and NIT-GNR **84**. (b) Multifrequency ESR spectra for NIT-polyphenylene **83** (green) and NIT-GNR **84** (red), along with simulations (black), plotted against the magnetic field from the edge-state resonance. Reproduced with permission from ref (221). Copyright 2018 Springer Nature.

Before looking at spin-bearing GNRs,^98^ whose synthesis has been described in section 5, another theoretical GNR study, which is relevant to device application, should be mentioned. This is the response of a GNR to external effects such as a transverse electrical field. This can induce the transition of a semiconductor to a semimetal and thus furnish Dirac Fermions in a semiconductor.^274^ Returning to molecular control, the NIT-GNR **84** (Figure 15a–b) carrying delocalized spins without having a zigzag edge has been subjected to time-resolved ESR spectroscopy. These experiments, which were performed in the group of Lapo Bogani, describe the evolution of a spin and, above all, yield ultrahigh spin coherence times in the microsecond range even at room temperature.^221^ These results were obtained for a stable material under ambient conditions and hold enormous promise for quantum operations executed by single-electron transport combined with electrical detection of spins.

**Figure 16:**
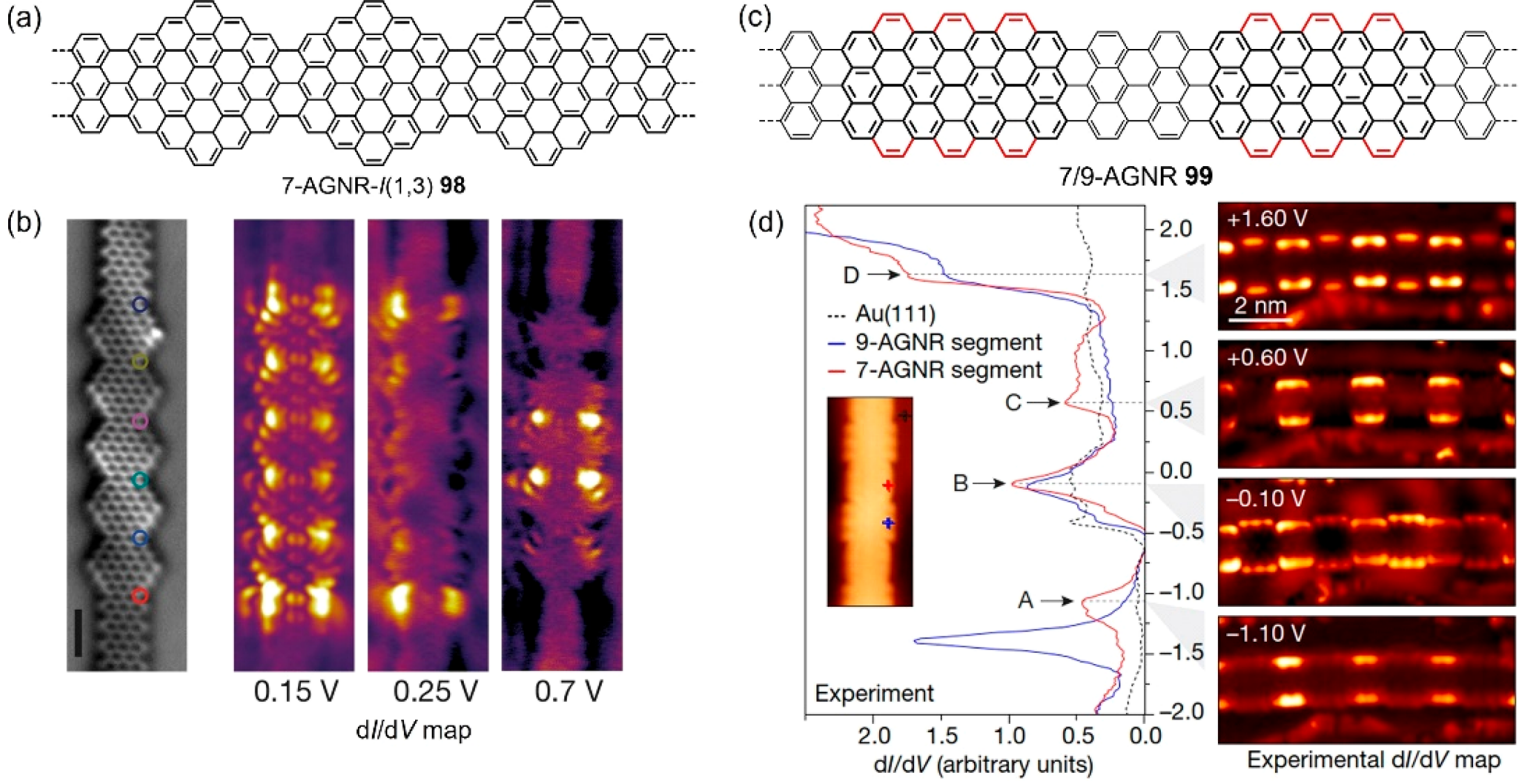
Chemical structures of (a) 7-AGNR-*I*(1,3) **98** and (c) 7/9-AGNR **99** exhibiting topological electronic phases. (b) Constant-height nc-AFM image and experimental d*I*/d*V* maps of 7-AGNR-*I*(1,3) **98** on Au(111). (d) d*I*/d*V* spectra taken at the locations indicated by the corresponding color markers, and constant-current d*I*/d*V* experimental maps of 7/9-AGNR **99** on Au(111). Reproduced with permission from refs (30, 31). Copyright 2018 Springer Nature.

It is fascinating that the topics of greatest interest in condensed matter physics are closely connected with syntheses of robust, but atomically precise GNRs possessing different edge structures. The reason is obvious: chemistry offers the potential of even engineering topological electronic phases and this provides access to exotic quantum states that are essential in spintronics or quantum computing. The theoretical basis of this concept is provided by the Su–Schrieffer–Heeger model,^275^ which has been extended from the classical description of polyacetylene to that of GNRs (Figure 16a) such as edge extended 7-AGNR-*I*(1,3) **98**.^30^ STS can verify controlled periodic coupling of topological boundary states and thus prove the existence of quasi-1D quantum phases (Figure 16b). The recent literature provides many similar cases of monitoring the rise of topological properties by material synthesis,^31,98^ such as GNR-based topological insulators.^276^ The 7/9-AGNR **99** are fabricated by partially adding K-regions to the armchair edge of 7-AGNRs (Figure 16c–d) to manifest nontrivial 1D topological phases.^31^ Thereby, important additions to structural control of GNRs, apart from width and edge, are syntheses of semimetallic chiral GNRs.^211^

Uncovering the many electronic functions of NGs and GNRs has led the community a long way. What begins with opening of a band gap and the associated engineering finds its highlights with the creation of exotic quantum states and new opportunities for physics, but physics, in turn, must now put these molecules to work.

## 8 Synthetic Carbon Materials in the World of Biology

The common belief that synthetic carbon nanostructures are strictly “nonnatural” can be easily proven wrong with their powerful roles in many bioapplications. Dendrimers have often been employed as molecularly defined “functional nanoparticles”.^277−279^ Therefore, carbon-rich polyphenylene dendrimers (PPDs) not only serve as precursors for syntheses of NGs but also stand out due to their shape-persistent arms.^280^ This structural feature, which results from the presence of stiff polyphenylene chains, promises precise nanosite definition when functional groups such as chromophores are placed at the core, the scaffold, or the surface.^281,282^ An example of outstanding biological importance is peripheral functionalization with alternating “patches” of polar and nonpolar groups. The semirigidity prevents clustering as a result of conformational changes within the dendrimer interior. This alternating array of polar and nonpolar functions furnishes solubility in both aqueous and organic solvents with unique cell permeability.^283,284^ Another surprising result is the aggregation behavior seen with charged particles such as viruses. Adenoviruses, for example, which are considered vectors for DNA transfection, can be decorated for uptake into tumor cells by aggregation with patched PPDs.^285^

Functionalized PPDs can also serve as building blocks for the assembly process. Examples are layer-by-layer deposition of PPDs with oppositely charged surfaces or nuclear staining using electrolyte-electrolyte and histone interactions to form thin films for high-sensitivity DNA detection.^286−289^ PPDs with peripheral thiomethyl groups give rise to ordered and porous gold nanoparticle–dendrimer composites in the solid state, and they are used in sensor applications due to their strong plasmon band.^290^ These accomplishments are rooted in the combination of advanced synthesis and the versatility of functionalized PPDs in tailoring their self-assembly properties. Similarly, networks can be formed from CNTs and dodecyl-substituted HBCs or other discotics to yield chemiresistors and cross-reactive arrays, which display excellent discrimination between volatile organic compounds in exhaled breath and serve as cost-effective, portable, and noninvasive diagnostic tools for detecting cancer and neurodegenerative diseases.^291−293^

**Figure 17:**
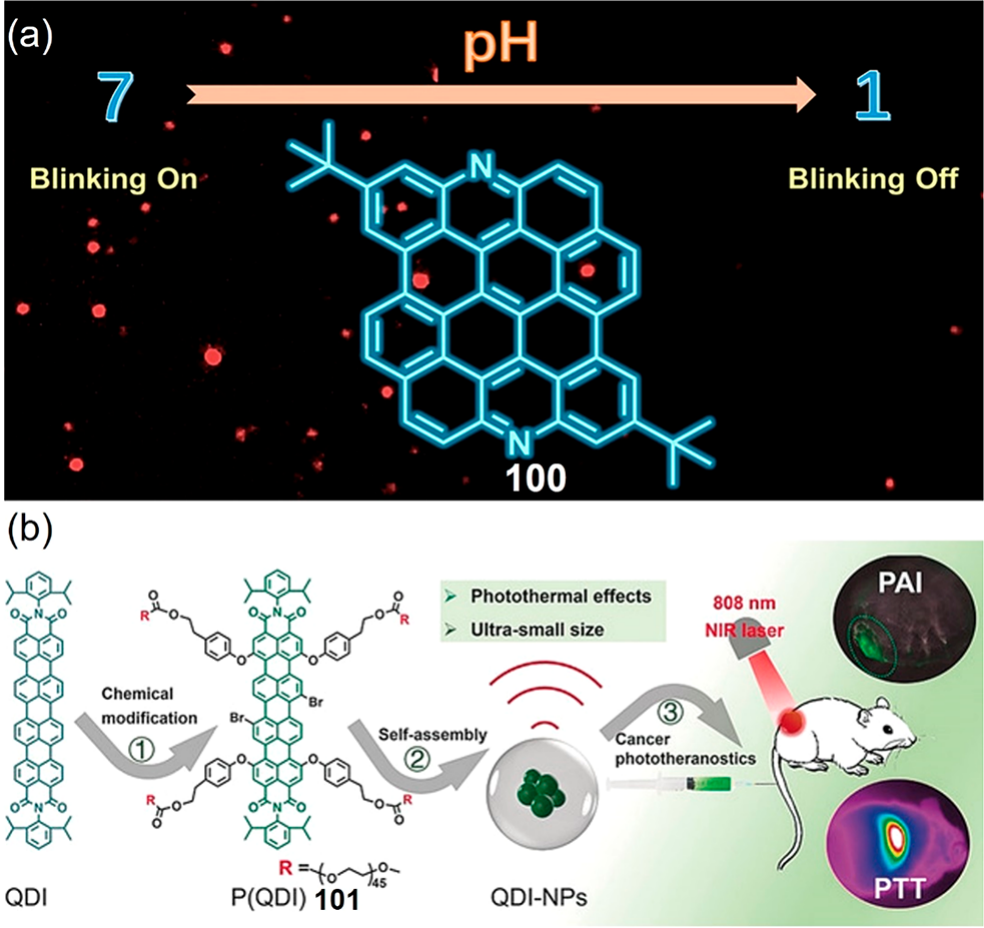
(a) N-doped DBOV **100** with pH-dependent blinking properties. Reproduced with permission from ref (294). Copyright 2021 American Chemical Society. (b) Self-assembling QDI-NPs with high-resolution photoacoustic imaging and efficient photothermal cancer therapy. Reproduced with permission from ref (295). Copyright 2019 John Wiley and Sons.

Precise control of photophysical and biological properties such as DNA-binding of synthetic NGs has provided a great variety of dyes as reporter molecules and imaging agents for biomedicine.^240,296−298^ For example, inspired by the excellent photophysical properties of DBOV **91** discussed in section 6, selective nitrogen-doping was achieved to produce N-DBOV **100** with strong luminescence (Figure 17a). Due to the presence of nitrogen atoms, N-DBOV exhibits a surprising pH-responsive blinking effect which enables pH-sensitive superresolution imaging.^294^ Another example is the important area of cancer theranostics realized by the combination of diagnosis (luminescence and photoacoustic imaging) and therapy (chemical, photodynamic, and photothermal therapy) using rylenecarboximides derivatives. The poly(ethylene glycol)-functionalized quaterrylenediimide (P(QDI), **101**) self-assembles into QDI-nanoparticles (QDI-NPs) with sizes of approximately 10 nm in aqueous solution (Figure 17b), which allows high-resolution photoacoustic imaging and efficient photothermal cancer therapy upon NIR laser irradiation.^295^ Further improvement of penetration depth, realization of rapid and early diagnoses of some critical diseases, and targeted therapy can well be expected in the future.

Porous graphenic nanostructures and other 2D materials have attracted considerable attention for sieving or sensing applications, including potential nucleic-acid sensing and DNA sequencing.^299−301^ It has been theoretically demonstrated that DNA nucleobases inserted into the nanopores of GNRs lead to unique changes in device conductance and allow DNA sequencing.^302^ While such biosensing devices have been proven experimentally with top-down preparation of a nanopore GNR with undefined edge structures,^303^ measurement sensitivity can be drastically enhanced by using zigzag-edged or topologically insulating GNRs,^302^ which are the synthetic challenges to be approached in the future.

Applications of GNRs in biological systems rely mainly on their luminescence and the presence of substituents for further functionalization. Grafting of polyamide or polyethylenimine (PEI) chains to GNRs delivers conjugates serving as carriers for gene therapy because of their notable affinities and specificities toward somatic cells and proteins.^304^ A remarkable study involved PEI-grafted GNR (PEI-*g*-GNR) as an effective gene vector for the locked nucleic acid modified molecular beacon (LNA-m-MB). The large surface area and high charge density of PEI-*g*-GNR protect the LNA-m-MB probes from nuclease digestion or binding interactions with proteins. The resulting complex LNA-m-MB/PEI-*g*-GNR leads to high transfection efficiency, which is favorable for sensitive detection of the recognized target microRNA, implying potential applications in gene therapy.^305^ The challenges for use of GNRs in the biomedical field can thus be summarized as reproducibility of the material, higher drug loading capacity, and lower toxicity, but all of these require controllable and atomically precise syntheses of GNRs.

## 9 New Opportunities in Energy Technologies

**Figure 18:**
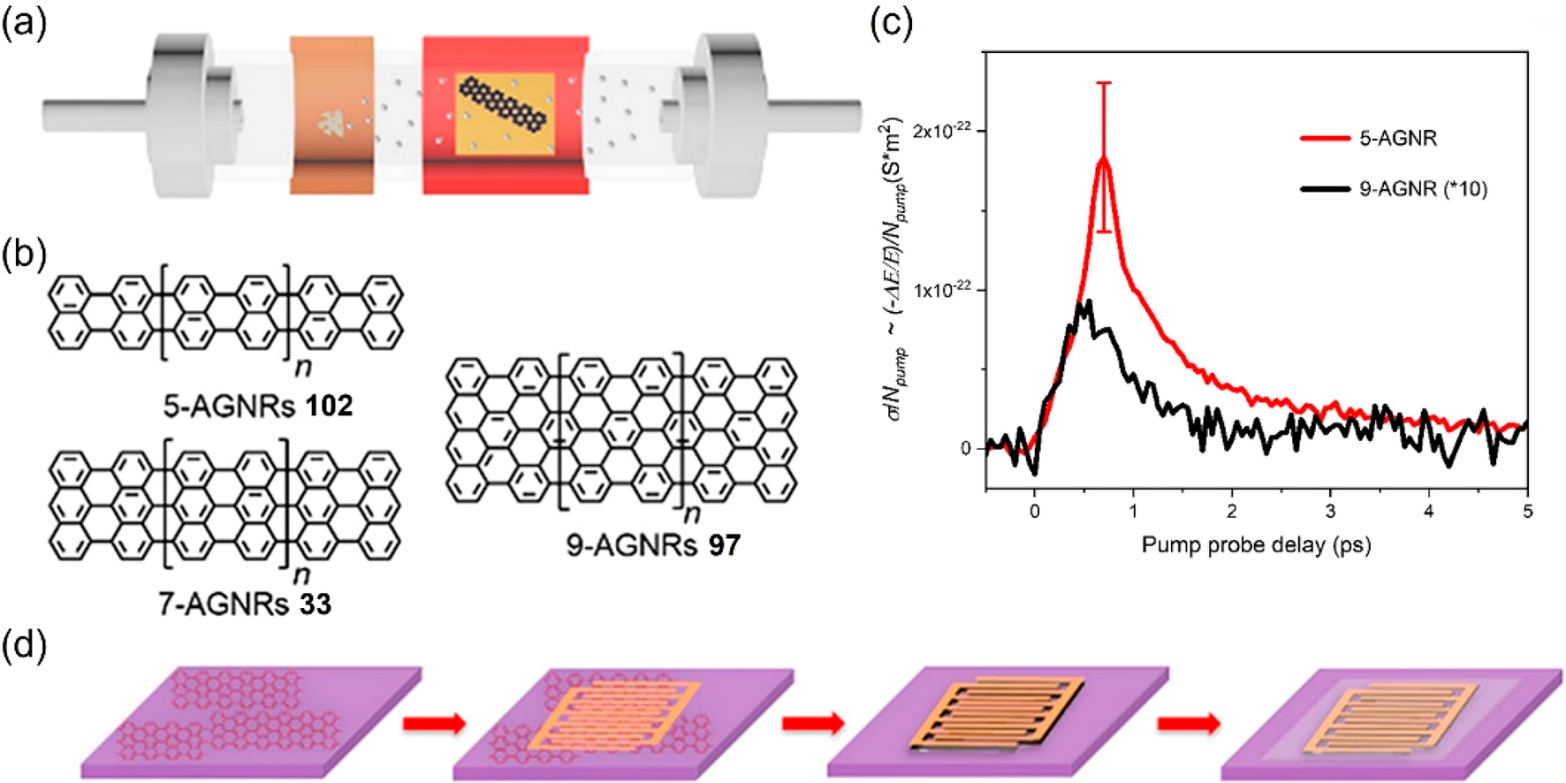
(a–b) Schematic illustration of the growth of 5-AGNR **102**, 7-AGNR **33**, and 9-AGNR **97** via CVD. (c) Time-resolved complex terahertz photoconductivity of 5-AGNR **102** and 9-AGNR **97** normalized to the absorbed photon density. (d) Schematic illustration for device fabrication of microsupercapacitors based on GNR films. Reproduced with permission from ref (310). Copyright 2020 American Chemical Society.

A common way of thinking in carbon nanostructure research is centered around the role of NGs and GNRs as “graphene models” whose sizes are increased to allow them to approach the behavior of graphene. This is probably missing the most important feature that was already discussed above: GNRs offer properties that graphene does not.^106^ This claim can be illustrated in the domain of energy technologies. What is needed for charge storage is high capacitance, high charging–discharging rates, and long cycling life.^306^ The layered structures of graphene, which are often fabricated as hybrids with conducting polymers, are particularly well suited for energy storage in supercapacitors due to the rapid influx of counterions.^307^ Indeed, graphenes obtained by electrochemically assisted exfoliation of graphite possess high energy densities and high power densities according to their Ragone plots.^308,309^ Moreover, GNRs prepared via a bottom-up CVD approach are superior to graphene with its large basal plane.^8^ Employing 5-, 7-, and 9-AGNRs as electrode materials for microsupercapacitors gives an excellent volumetric capacitance of 307 F cm^–3^ and ultrahigh power densities of up to 2000 W cm^–3^, and the narrowest ribbon is the best. This electrochemical performance of microsupercapacitors can be rationalized by the largely increased edges and the high charge-carrier mobilities, as determined by pump–probe terahertz spectroscopy (Figure 18).^310^

“Segmenting” the graphene sheet is a matter of not only cutting out smaller subunits but also avoiding stacking, and that is why obstructing aggregation of GNRs is equally important before putting them to work in charge storage. Alkyl substituents can improve the solubility of NGs and GNRs, but they also “dilute” desired electronic properties with regard to applications in charge-storage devices.^311^ GNRs without solubilizing groups have also been prepared in solution on a gram scale, but these insoluble GNRs can only be processed by strong sonication, which is known to break them down and shorten their lengths.^58,312,313^ Therefore, although it may not appear too appealing from the perspective of atom economy, thermal removal of alkyl chains after film formation has become an established protocol.^268^

**Figure 19:**
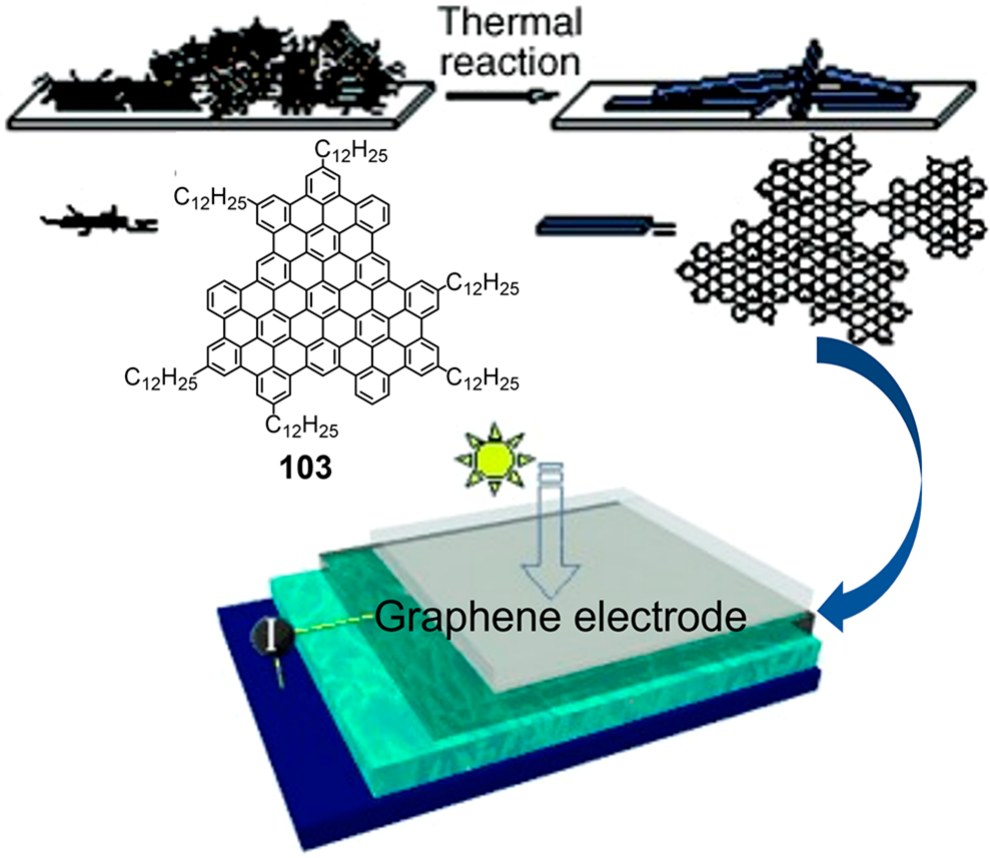
Bottom-up chemical approach used to synthesize a transparent graphene film from superphenalene derivative **103** and application of the graphene as a window electrode in an organic solar cell. Reproduced with permission from ref (314). Copyright 2008 John Wiley and Sons.

To realize practical applications in energy technologies, however, one may not need precisely defined edges. Instead, robust, high-yielding syntheses and cheaper processing protocols would be in urgent demand. Understandably, therefore, top-down approaches, including lithographic cutting of graphene, sonochemical treatment of graphite in solution, unzipping of CNTs, and most importantly, pyrolysis, have been widely applied to prepare graphenic nanostructures.^315−317^ From the perspectives of organic and polymer chemistry, pyrolytic processes may be considered undefined techniques with mostly unknown reaction mechanisms, but they are of immense value when, for example, encapsulating metal, metal oxide, or silicon particles for morphologically stable anode materials of batteries.^318^ Producing graphite or carbon nanofibers from asphalt-like solid pitches is a major industrial challenge.^319,320^ One inspiring case is the phase-forming superphenalene derivative **103**, which is spin-coated on substrates and then heated at 1100 °C in an argon atmosphere.^321^ The resulting conductive carbon films are transparent over the wavelength range 260 to 800 nm^322^ and thus offer suitable “window electrodes” for photovoltaic or electroluminescent devices (Figure 19).^314^ Fabrication of large-scale and ultrathin graphene films by CVD or reduction of graphene oxide has also been employed toward that end,^323,324^ but spin-coating of large, yet soluble, NGs and subsequent pyrolysis of the NG films at temperatures above 1000 °C offer a unique alternative.^325^

**Figure 20:**
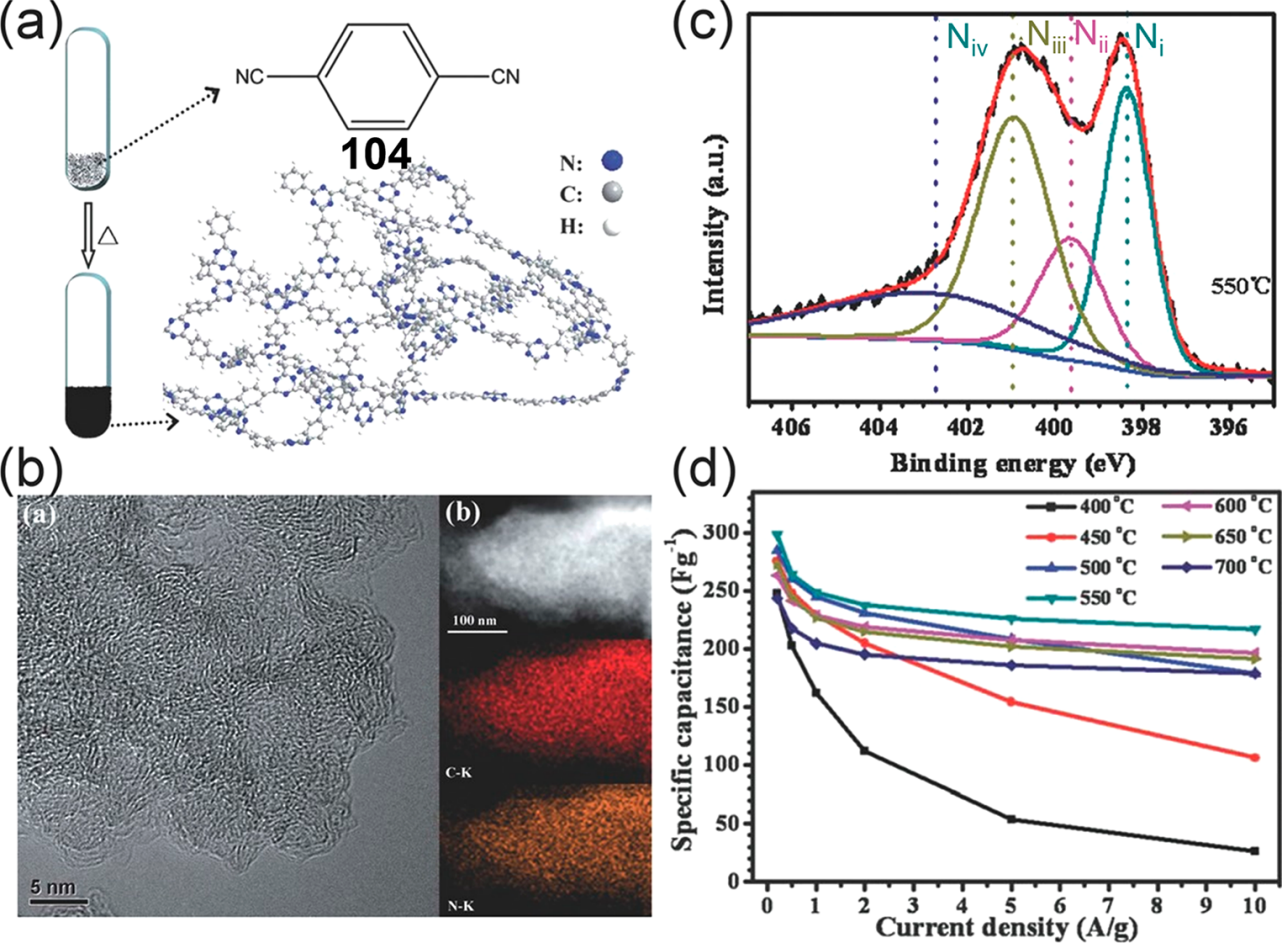
(a) Ionothermally induced synthesis of TNNs from terephthalonitrile **104**. (b) High-resolution TEM and STEM images of TNNs. (c) Typical N 1s XPS spectrum of TNNs with four distinct nitrogen configurations. (d) Specific capacitances at various current densities. Reproduced with permission from ref (328). Copyright 2012 Royal Society of Chemistry.

Ionothermally induced polymerization of small aromatic molecules at a high temperature can also lead to formation of porous polymer networks with high surface areas, adjustable pore sizes, controllable chemical structures, and adaptable chemical functionalities.^326,327^ Treatment of terephthalonitrile **104** at 400 °C provides a covalent triazine-based framework with poor electrical conductivity. Heating to 550 °C, however, produces nitrogen-rich networks (TNNs) serving as high-performance electrode materials for supercapacitors (Figure 20).^328^

What these few energy applications point out is that economically feasible protocols and more demanding syntheses can well go hand in hand.

## 10 Conclusions and Outlook

NGs and GNRs, the title structures of this article, highlight the enormous versatility of organic chemistry in realizing π-conjugation, which also stands at the origin of organic electronic materials due to their ability to interact with light and undergo electron transfer. The prototypes of π-conjugation are oligoenes, their cyclic congeners (known as annulenes), and simple PAHs. Further, fundamental features such as the HOMO–LUMO energy gap, orbital symmetry, or orbital degeneracy have proven to be indispensable tools in understanding or even predicting material properties. Currently, an increasing number of theoretical models are used to demonstrate the electronic and quantum properties of NGs and GNRs, such as the occurrence of topological phases; this, in turn, stimulates design of more unprecedented graphenic molecules. The current literature on NGs and GNRs offers ample examples of the lively interplay of theory and experiment.^329,330^

When considering a homologous series of oligomers, polyacetylene might appear as the logical end point for extending oligoene chains. However, what is theoretically enlightening is much less straightforward in synthesis. Oligoenes, when made in solution, appear to be extremely unstable, while the corresponding “polyene”, namely, polyacetylene, has been obtained by catalyzed polymerization of acetylene as a solid and thus profited from its lattice energy.^331^ There is thus no trivial connection between small conjugated molecules and their related polymeric materials. While Geim’s and Novoselov’s ingenious experiment on peeling off graphene flakes from graphite with scotch-tape has opened the groundbreaking world of 2D electronic materials,^253,332−335^ this approach does not lay the ground for robust fabrication protocols, nor does continuous extension of NGs create a feasible transition to graphene materials. An important additional aspect is that many conjugated polymers in the early science and technology of “synthetic metals” excelled due to their tremendous increase of electrical conductivity upon doping,^336^ but not from their structural precision. Structural precision, however, is a key requirement when targeting unconventional properties of GNRs.

These GNRs establish a class of quasi-1D semiconductors which may close the gap between linear conjugated polymers and graphene. GNRs offer ample opportunities for band gap and bandwidth engineering, which can control the performance of electronic devices. However, the chain-type structures of conjugated polymers allow band gap engineering as well, and low band gap polymers have been long sought targets. GNRs have the potential of maintaining the high charge-carrier mobilities of graphene while at the same time furnishing a finite band gap. Nevertheless, the real value of GNRs lies in the formation of exotic quantum states as the starting point for future quantum technologies. The underlying principles may well go beyond the capacity of a chemist, but these exciting features, such as the creation of zigzag edges, defined defects, and nonplanarity, are intimately connected to the power of chemistry. What is needed, therefore, is a more general concept of synthesis in the future. Zigzag edges or high-spin structures may not persist when formed in solution or under ambient conditions, thus requiring the stabilization of a metal surface or demanding UHV conditions. Therefore, keeping spins apart for “stabilization” will conflict with the need for strong magnetic exchange coupling. In view of future defect engineering, the most promising directions may emerge from the precise synthesis of NGs and GNRs embedding pentagon–heptagon pairs, which offer a whole new landscape of planar yet, nonbenzenoid carbon structures with unique properties. When nonplanarity is introduced, such as in helicenes and cyclacenes,^337^ the NGs and GNRs can “rise” from flatland with interesting chiral optoelectronic perspectives. Due to their macromolecular character and limited solubility, development of more advanced characterization methods for ultralarge NGs and GNRs is necessary. The importance of solid structures and their packing modes has been emphasized above, but many applications of NGs and GNRs require deposition on insulating substrates, and adequate transfer procedures are another need for future device fabrication.

Tightly connected with this refined understanding of materials synthesis is its combination with nanoscience. Synthesis of NGs and GNRs on surfaces goes far beyond visualization and *in situ* structure characterization, as was convincingly proven by detection of topological phases via STS or determination of magnetic exchange coupling through inelastic electron tunneling spectroscopy.^338^

Other carbon nanostructures such as nanodiamonds also open breakthroughs in physics as well as life science.^339,340^ Similarly, the above applications of NGs and GNRs in sensing, diagnostics, and therapy originate from fundamental electronic properties and their subtle dependence upon assembly and interfacing processes. On the other hand, the growing roles of NGs and GNRs as multitalents of physics and life sciences by no means imply that the demanding chemistry of NGs and GNRs has come to an end. Thus, controlling the multihelicity of larger and larger graphenic molecules is still in its infancy and the importance of porphyrins and phthalocyanines as chromophores and redox units strongly suggests their incorporation into growing graphene honeycomb molecules. Perfluorination of NG-edges or perhydrogenation of NGs toward nanographanes would be further challenges. In view of practical applications, upscaling of bottom-up synthesis is critically important, and minimum reaction steps and low-cost reagents should be considered.

Another question is whether fine-tuning of electronic structures will always require a new bottom-up synthesis. A good case has been made above by covalently attaching small molecules such as chromophores or stable free radicals to the periphery of parent NGs and GNRs to allow exciton or spin transfer. Beyond covalent bonding, small π-conjugated molecules can be deposited on GNRs to modify their electronic structures and achieve ordered supramolecular arrangements. The above-mentioned porphyrins or phthalocyanines offer defined anchor points for binding of guest molecules and, thus, out-of-plane growth. Likewise, while holey graphenes have found much attention, similar approaches hold enormous promise for NGs and GNRs, and selective binding of guest molecules in holes or at heteroatoms will be increasingly adopted for sensing purposes.

GNRs are available not only from sophisticated syntheses but also from harsh top-down methods. One is tempted to emphasize the need for precision synthesis in producing, for example, unobstructed edge structures or defined defects. There are, however, materials properties that may not need this high precision, thus allowing more relaxed and cheaper methods. Independent of how they are made, with their unprecedented properties, NGs and GNRs now emerge as components of new technologies. This point, however, would have never been reached without pioneers such as Erich Clar and Erich Hückel, who recognized the fascinating structure–property relationships of basic conjugated molecules.
